# What Is the Weather Prediction Task Good for? A New Analysis of Learning Strategies Reveals How Young Adults Solve the Task

**DOI:** 10.3389/fpsyg.2022.886339

**Published:** 2022-06-13

**Authors:** Emilie Bochud-Fragnière, Pamela Banta Lavenex, Pierre Lavenex

**Affiliations:** ^1^Laboratory of Brain and Cognitive Development, Institute of Psychology, University of Lausanne, Lausanne, Switzerland; ^2^Faculty of Psychology, UniDistance Suisse, Brig, Switzerland

**Keywords:** probabilistic learning, multiple-cue learning, conditional learning, hippocampus, striatum, explicit, implicit

## Abstract

The Weather Prediction Task (WPT) was originally designed to assess probabilistic classification learning. Participants were believed to gradually acquire implicit knowledge about cue–outcome association probabilities and solve the task using a multicue strategy based on the combination of all cue–outcome probabilities. However, the cognitive processes engaged in the resolution of this task have not been firmly established, and despite conflicting results, the WPT is still commonly used to assess striatal or procedural learning capacities in various populations. Here, we tested young adults on a modified version of the WPT and performed novel analyses to decipher the learning strategies and cognitive processes that may support above chance performance. The majority of participants used a hierarchical strategy by assigning different weights to the different cues according to their level of predictability. They primarily based their responses on the presence or absence of highly predictive cues and considered less predictive cues secondarily. However, the influence of the less predictive cues was inconsistent with the use of a multicue strategy, since they did not affect choices when both highly predictive cues associated with opposite outcomes were present simultaneously. Our findings indicate that overall performance is inadequate to draw conclusions about the cognitive processes assessed by the WPT. Instead, detailed analyses of performance for the different patterns of cue–outcome associations are essential to determine the learning strategies used by participants to solve the task.

## Introduction

The Weather Prediction Task (WPT) was originally designed to assess probabilistic classification learning (Knowlton et al., 1994). In this task, four cues are associated probabilistically with different outcomes and these associations are assumed to be established without requiring the participants’ explicit awareness. Typically, participants are told that they will forecast the weather in an imaginary city by learning to predict whether there will be sunshine or rain by taking into consideration several different cues. The cues are four different tarot cards, and either one, two, or three of these cards can be shown on any given trial, thus generating 14 unique combinations or patterns of cues. On each trial, the pattern of cues is presented with the two possible outcomes (i.e., sunshine or rain), and participants must predict whether the outcome is more likely to be sunshine or rain. Importantly, just like the weather, the relationships between individual cues and outcomes are probabilistic and thus not perfectly correlated. Specifically, one highly predictive cue is associated with rain on 75% of the trials and with sun on the remaining 25%, whereas the other highly predictive cue is associated with sun on 75% of the trials and rain on the remaining 25%. In contrast, the two less predictive cues are associated with rain (or sunshine) on 57% of the trials and with the opposite outcome on the remaining 43%. Thus, on a given trial, some cues may be associated with a less probable outcome. Researchers originally reasoned that the probabilistic nature of the WPT should hinder direct explicit memorization of the cue–outcome associations (Knowlton et al., 1994). Instead, it was hypothesized that participants gradually acquire implicit knowledge about cue–outcome associations over many trials using trial-and-error feedback. The double dissociation found between amnesic and Parkinson’s disease patients during early training supported this hypothesis (Knowlton et al., 1994, 1996a), and led the authors to conclude that early learning could occur in absence of declarative knowledge and that the striatum was implicated in acquiring the non-motor habits necessary to solve the task.

Although several studies have questioned the view that the WPT implicates primarily non-declarative learning and memory processes (Knowlton et al., 1996a; Poldrack et al., 2001; Gluck et al., 2002; Hopkins et al., 2004; Foerde et al., 2006, 2007; Lagnado et al., 2006; Meeter et al., 2006; Newell et al., 2007; Price, 2009; Li et al., 2016), numerous studies evaluating the learning capacities of various clinical populations were conceived with the notion that the WPT assesses striatum-dependent or procedural learning. Indeed, the WPT or the Ice Cream Task, a modified version of the WPT in which participants must predict the flavor of ice cream preferred by a cartoon figure based on the different accessories worn by that figure (Shohamy et al., 2004a), have been extensively used in clinical populations to evaluate the behavioral consequences of known or presumed structural or functional basal ganglia deficiencies: for example in schizophrenia (Keri et al., 2000; Weickert et al., 2002; Foerde et al., 2008; Horan et al., 2008; Gomar et al., 2011; Karcher et al., 2019; Fernandez et al., 2021), Tourette syndrome (Keri et al., 2002; Marsh et al., 2004), bulimia nervosa (Labouliere et al., 2016), attention-deficit hyperactivity disorder (Gabay and Goldfarb, 2017), Parkinson and Huntington diseases (Knowlton et al., 1996a,b; Shohamy et al., 2004a,b), and children with acquired or developmental basal ganglia pathology (Mayor-Dubois et al., 2010). Researchers have also used these tasks to assess memory capacities in individuals with neurodevelopmental disorders thought to be characterized by some procedural memory deficits, such as autism (Brown et al., 2010; Obeid et al., 2016), obsessive–compulsive disorder (Exner et al., 2014; Kelmendi et al., 2016; Hansmeier et al., 2018), specific language impairment (Kemeny and Lukacs, 2010; Mayor-Dubois et al., 2014; Obeid et al., 2016), or developmental dyslexia (Gabay et al., 2015). However, to properly interpret the results of investigations employing these tasks, it is critical to precisely define the cognitive processes actually engaged during their resolution, and not rely simply on the fact that overall task performance may be more or less impaired in individuals with certain pathologies.

Accordingly, in addition to identifying which brain structures may contribute to overall performance in the WPT, researchers have also attempted to identify the possible learning strategies individuals may use to solve the WPT. Originally, participants were assumed to solve the WPT uniquely using what is known as a multicue strategy: predicting the correct outcome based on the combined probability of all the cues (Knowlton et al., 1994). However, further research has shown that strategies other than the multicue strategy may be used to solve the WPT. In particular, Gluck et al. (2002) developed new analysis methods to define the possible strategies used by each individual to solve the WPT and thus decipher the cognitive processes enabling successful task performance. They compared the actual responses of each participant to theoretical models of responses that would be obtained if participants strictly followed one of the four strategies that the participants themselves reported to have used: (1) the *multicue* strategy in which participants answer correctly on every trial, that is perfect performance, as defined by the experimenters; (2) the *singleton* strategy in which participants only give correct answers for trials with one single cue, and respond randomly when more than one cue is presented; (3) the *one-cue (highly predictive)* strategy in which participants base their responses on the presence or absence of one of the two highly predictive cues, even when other cues are present; or (4) the *one-cue (less predictive)* strategy in which participants base their responses on the presence or absence of one of the two less predictive cues, even when other cues are present. For each participant, the strategy with the lowest difference score (i.e., the difference between actual responses and theoretical responses under an arbitrary criterion that enabled the authors to propose a specific strategy for 59/60 participants) was considered the strategy used by that participant. Their analyses suggested that different participants may use different strategies to solve the WPT. Moreover, analyses by blocks of 50 trials suggested that some participants may change their strategy over the course of the task. Finally, in contrast to what was originally hypothesized, this analysis suggested that the majority of participants did not use the multicue strategy to solve the WPT. Although different participants used different strategies, a majority used the singleton strategy, in particular during the first 100 trials. However, the authors also reported that there was an increase in the number of participants using the one-cue strategy and the multicue strategy after the first 100 trials. Importantly, although the participants’ subjective impressions had been used to define the four strategies used to solve the task, their impression did not necessarily coincide with the strategy identified based on the best correspondence between their actual responses and the theoretical models.

In a subsequent study, Meeter et al. (2006) introduced another method for analyzing the data and identified even more possible strategies that could be used to perform the WPT: (1) The *random* strategy, which is defined by the probability of random choices for all patterns. This strategy was considered to best describe the performance of participants who, for example, did not follow a strategy, switched between strategies, or used an unidentified strategy (e.g., alternating between left and right outcomes). The authors argued that in Gluck et al. (2002), such participants were incorrectly classified as using the singleton strategy. (2) The *singleton strong* strategy, in which participants learn how the patterns containing only one highly predictive cue predict the outcome. (3) The *singleton* strategy (already described in Gluck et al. (2002)), in which participants learn how the patterns containing only one cue predict the outcome. (4) The *singleton + prototypes* strategy, in which participants learn both how patterns with one single cue, and patterns combining two congruent cues predict the outcome. (5) The *2* *vs. 1* strategy, in which participants learn how patterns with one single cue, patterns combining two congruent cues, and patterns combining two congruent cues and one incongruent cue predict the outcome. In contrast to the multicue strategy, however, participants attribute the same weight to all cues, meaning that the less predictive cues are considered by the participant to be as reliable as the highly predictive cues. (6) The *all but two strong cards* strategy, in which participants learn the correct answer for all the patterns except when both highly predictive cues are present at the same time. (7) The *perfect* strategy (defined as the multicue strategy in Gluck et al. (2002)), in which participants learn the correct answer for all possible patterns. (8) The *single-cue strong* strategy (defined as the one-cue highly predictive strategy in Gluck et al. (2002)), in which participants attend selectively to one highly predictive cue (i.e., the presence or absence of one highly predictive cue associated with either sun or rain) and ignore the other cues. (9) The single-cue weak strategy (defined as the one-cue less predictive strategy in Gluck et al. (2002)), in which participants attend selectively to one less predictive cue (i.e., the presence or absence of one less predictive cue) and ignore the other cues.

Finally, Meeter et al. (2006) also identified inflection points that signaled the shift between two different strategies by the same participant, ostensibly improving the precision with which they were able to attribute reliance on a particular strategy by an individual participant. However, careful examination of their analysis methods reveals an important limitation regarding their underlying assumptions. Specifically, Gluck et al. (2002) and Meeter et al. (2006) considered that the strategy with the lowest score under an arbitrary criterion of 0.1 was used by the participant. However, no statistical comparison provided evidence that other strategies with similar scores yielded inferior results since their significance cannot be tested at the individual level.

In sum, despite the fact that several studies have shown that the WPT does not necessarily assess striatal-dependent implicit memory in healthy young adults (Knowlton et al., 1996a; Poldrack et al., 2001; Gluck et al., 2002; Hopkins et al., 2004; Foerde et al., 2006, 2007; Lagnado et al., 2006; Meeter et al., 2006; Newell et al., 2007; Price, 2009; Li et al., 2016), this task is still used to determine whether specific populations exhibit impairments of this memory system (Knowlton et al., 1996b; Keri et al., 2000, 2002; Weickert et al., 2002; Marsh et al., 2004; Shohamy et al., 2004a,b; Foerde et al., 2008; Horan et al., 2008; Brown et al., 2010; Kemeny and Lukacs, 2010; Mayor-Dubois et al., 2010, 2014; Gomar et al., 2011; Exner et al., 2014; Gabay et al., 2015; Kelmendi et al., 2016; Labouliere et al., 2016; Obeid et al., 2016; Hansmeier et al., 2018; Karcher et al., 2019; Fernandez et al., 2021). Moreover, knowing that different learning strategies can lead to above chance performance in the WPT, it is fundamental to determine how participants solve the task (Ashby and Maddox, 2005). Unfortunately, the strategy analyses proposed by Gluck et al. (2002) and Meeter et al. (2006), requires exhaustive *a priori* identification of the different strategies that may be used to perform the WPT, leading to the possibility that participants who use a non-considered strategy will be improperly classified. Thus, to more conclusively identify the learning strategies and cognitive processes engaged when solving the WPT, it is necessary to evaluate the performance of participants on individual patterns of cues to demonstrate exactly which cue–outcome associations have been learned and the influence of the different categories of cues (highly vs. less predictive) on the participants’ choices.

Here, we tested 22 healthy young adults with a modified version of the WPT originally designed by Knowlton et al. (1994, 1996a,b). We present new analyses designed to identify the learning strategies and cognitive processes supporting above chance performance in the WPT. First, we adopted the strategy analysis methods of Gluck et al. (2002) and Meeter et al. (2006) to analyze performance at the individual and group levels. We tested all of the strategies identified by Gluck et al. (2002) and Meeter et al. (2006) as well as a previously overlooked additional strategy: the *two most predictive cues* strategy. Second, we implemented a novel analysis to evaluate how participants performed on each unique pattern of cues (i.e., the 14 unique combinations of the four cues) to determine whether participants who succeeded on the task performed as would be predicted by each strategy for each pattern. In contrast to the strategy analyses designed by (Gluck et al., 2002; Meeter et al., 2006), which can only propose a strategy that is likely used to solve the WPT but cannot exclude the use of alternative strategies, our new analyses can define the strategy used and exclude alternative strategies at the group level.

## Materials and Methods

### Participants

Participants were 22 healthy young adults (11 females; average age: 26.29 years; range: 21.72–30.82) of middle-class socio-economic status. They were recruited in Switzerland *via* personal connections. Exclusion criteria were atypical development (e.g., developmental delay or high potential), neurological disorders (e.g., epilepsy), or chronic substance use (drugs or medications). Testing took place Monday through Saturday, between 11:00 AM and 9:00 PM. Participants were generally tested at their home or at the home of the experimenter. The only requirement for testing was a quiet room with a table and two chairs. All testing sessions were videotaped. Participants gave written informed consent prior to beginning the study and received a gift card to a local retailer for their participation. Human subjects research was approved by the Cantonal Ethics Commission for Human Research [Vaud, Switzerland; project no PB_2017-00074 (60/14)] and was in accordance with the code of ethics of the World Medical Association (Declaration of Helsinki) for experiments involving human subjects in research.

### General Procedure

**Figure 1:**
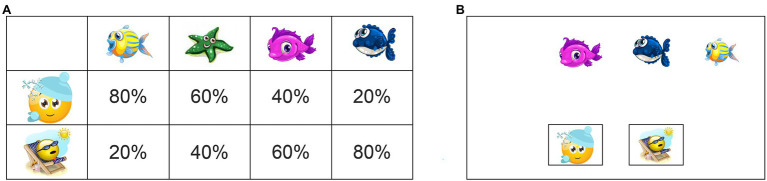
**(A)** Example of one set of four cues (sea animals), which were associated with each outcome (sun or snow) with a fixed level of probability across all patterns. **(B)** Representation of the computer screen for one trial when three animals were displayed, and the participant had to choose between snow and sun. Note that one, two or three animals could be displayed on any given trial; all four animals were never displayed simultaneously.

This new WPT was modified from the original WPT (Knowlton et al., 1994) and from the Ice Cream Task (Shohamy et al., 2004a) to test young children in future studies. First, cartoonish sea animals were used as cues instead of tarot cards, and two different smiley faces served as outcomes (Figure 1). Second, to give young participants a better chance to complete the task we used only 100 trials, and we modified the associative outcome probabilities to 0.20, 0.40, 0.60, 0.80, as was previously done by Gluck et al. (2002) and Mayor-Dubois et al. (2016). We therefore adapted the number of presentations of each pattern to respect these probabilities (Table 1). Our task was programmed with the E-Prime^®^ 3.0 software (Psychology Software Tools) and was run on a computer tablet with a tactile screen (HP Spectre × 360 Convertible 13-w0XX running with Microsoft© Windows^®^ 10 64-bit; Processor Intel Core i7; 8 GB RAM; 13,3-in (1,920 × 1,080); landscape orientation). The participants’ choice was recorded automatically by E-prime when they touched one of the two outcome stimuli (i.e., sun or snow).

**Table 1:** Number of occurrences of each pattern containing one to three cues, and their association with outcome A (snow) or B (sun).

	Cues				Outcome A	Outcome B	Total number ofoccurrences
Pattern	1	2	3	4	Snow	Sun	
1	0	0	0	1	8	1	9
2	0	0	1	0	4	1	5
3	0	0	1	1	12	1	13
4	0	1	0	0	1	3	4
5	0	1	0	1	5	1	6
6^a^	0	1	1	0	1	2	3
7	0	1	1	1	9	1	10
8	1	0	0	0	1	8	9
9^a^	1	0	0	1	2	2	4
10	1	0	1	0	1	6	7
11	1	0	1	1	2	1	3
12	1	1	0	0	1	12	13
13	1	1	0	1	2	3	5
14	1	1	1	0	1	8	9
TOTAL	7	7	7	7	50	50	100

a Patterns 6 and 9 do not have a correct answer because they combine either two cues that are highly predictive for both sun and snow (pattern 9) or two cues that are less predictive for both sun and for snow (pattern 6). They were not included in the analyses, which thus comprised 93 trials instead of 100. Note that the probability that each individual cue was associated with each outcome was calculated across all patterns. See main text for details.

### Stimuli and Detailed Procedure

We created four unique sets of four cues (cartoonish images of sea animals), and two images were used for the outcomes: a smiley face with sunglasses enjoying the sun (hereafter referred to as “sun”) and a smiley face wearing a knit hat enjoying the snow (hereafter referred to as “snow”; Figure 1; Supplementary Figure S1). One set of four cues was pseudo-randomly attributed to each participant. The WPT was divided into two types of trials: learning trials and test trials. In both parts, each trial was preceded by a fixation cross displayed for 1 s at the center of the screen. Subsequently, the stimuli appeared. During the learning trials, participants were required to choose which of two outcomes (sun or snow) was predicted by the combination of one to three cues (chosen from among the four sea animals that combined to produce 14 different patterns; Table 1). The instructions were the same for all participants: “You will see some fish, and you will have to discover if they prefer to play in the sun or in the snow.” Then, the experimenter or the participant touched the screen to begin the task. The order of the trials and the position of the cues (each cue could appear at one of four possible positions on the screen: left, center left, center right, right) were randomly determined with E-prime. The position of the sun and snow outcome images were fixed for all participants (Figure 1B).

Participants thus had to learn, using trial-by-trial feedback, which patterns were associated with which outcome. Once participants had indicated their choice by touching the screen, a congruent or incongruent outcome image appeared, depending on the predetermined probability associated with the cues (*NB*: the cues remained visible during this time). The experimenter also provided verbal feedback that was consistent with the visual feedback (“yes,” “right,” “no,” “it’s no big deal,” “not this one,” and “Oops, no”) until participants demonstrated the ability to understand the visual feedback (as evidenced when participants nodded their head or gave themselves verbal feedback). Therefore, incongruent trials could not be differentiated from congruent trials based on feedback, but rather only by cumulatively considering all the feedback given for each cue or pattern across trials. The participants then pressed a red button at the bottom of the screen to advance to the next trial. It is important to understand that a participant can make an optimal (correct) prediction but receive incongruent feedback. Thus, for example, even though fish X was correlated 80% of the time with sun, 20% of the time when fish X was shown, the snow outcome was displayed on the screen. During scoring the choice was judged as correct if it corresponded to the most probable outcome for that combination of cue(s), regardless of the displayed outcome image. For example, for pattern 0001, the appropriate prediction judged as correct is always outcome A (snow), even if in one out of five trials the feedback will be outcome B (sun; Table 1).

After 100 training trials, a four-trial test without feedback was presented during which each cue was displayed individually (patterns 0001, 0010, 0100, 1000) in the presence of the two possible outcomes (sun and snow), and participants were asked to choose which outcome was associated with each individual cue. Once participants indicated their choice by touching the outcome image on the screen, the next trial began with the fixation cross. The instructions for the test trials were the same for all participants: “You will see a fish on the computer screen and you will have to remember if it preferred to play in the snow or in the sun.” Then, the experimenter or the participant touched the screen to begin the test.

For each trial (during learning and test phases), the computer recorded the choice of the participant and the reaction time. Since reaction time depends on self-confidence and because limiting the time available to choose could induce impulsivity, the stimuli were presented for as long as needed for the participant to choose one outcome by touching the corresponding image. Nevertheless, although we did not impose a response time inferior to 5 s as was originally done (Knowlton et al., 1994, 1996a,b), our participants’ average response time (*M* = 2.60 s; SD = 0.55) was similar to that observed in previous studies. During the whole experiment, the experimenter sat next to the participant and wore sunglasses to preclude cueing participants with respect to the correct choice with their eye gaze.

### Data Analysis

#### Global Performance

All statistical analyses were performed with SPSS 27.0 software. We reported effect size with partial eta squared ($\eta_{p}^{2}$) for ANOVAs and Cohen’s *d_z_* for paired or one-sample *t*-tests. We used two-tailed paired samples *t*-tests to determine whether participants exhibited a bias toward choosing one of the two different outcomes, sun or snow, irrespective of the cues presented. As described in the results, we considered only 93 trials when calculating an overall performance score, since patterns 0110 (*n* = 3) and 1001 (*n* = 4) predicted both outcomes equally and were thus excluded from the analyses. We normalized the number of times outcome A (snow) and the number of times outcome B (sun) were chosen by dividing them with the number of correct “snow” and “sun” responses (i.e., 46 and 47, respectively; the difference is due to the overall distribution of cue and outcome combinations across all 100 trials). During training trials, participants did not choose “sun” (*M* = 1.03, SD *=* 0.10) more than “snow” (*M* = 0.97, SD *=* 0.10; *t_(21)_* = 1.606, *p* = 0.123, *d_z_* = 0.342). During test trials, participants did not choose “sun” (*M =* 1.95, SD = 0.67) more than “snow” (*M =* 2.05, SD = 0.67; *t_(20)_* = 0.326, *p* = 0.748, *d_z_* = 0.071; one participant did not perform the test trials). In sum, participants did not exhibit an overall bias toward choosing one of the two different outcomes irrespective of the cues. Since there was no effect of gender in any of the analyses performed (data not shown), data from men and women were combined for presentation.

Individual above chance performance during training was defined statistically with a chi-square test as 56 correct choices out of 93 ($\chi_{\left(1\right)}^{2}$ = 3.882, *p* = 0.049). To determine whether the entire group of participants performed above chance during training and test trials, we performed one-sample *t*-tests comparing the mean performance of the entire group of participants to the theoretical mean.

#### Modeled Strategies Analysis

As discussed in the introduction, participants may use several strategies to perform the WPT above chance level. Here, we describe in more detail the modified definitions of the nine strategies identified by Gluck et al. (2002; “G”) and Meeter et al. (2006; “M”; Table 2). Note that whereas Meeter et al. (2006) chose to define the strategies based on the results obtained for each pattern, we have tried to use definitions that are more representative of the cognitive processes associated to each strategy. This modified nomenclature does not impact what specific pattern of performance is expected for each strategy. The different strategies therefore included: (1) the *multicue* (G) or *perfect* (M) strategy, in which participants learn the correct answer for all the patterns by considering the probability of all cues simultaneously, as was originally proposed by Knowlton and colleagues. (2) The *all but two strong cards* strategy (M), in which participants base their responses on the presence or absence of the two highly predictive cues. However, when these cues are not present participants learn how the patterns containing only one of the less predictive cues predict the outcome. This strategy predicts chance performance for patterns 1011 and 1101, since participants rely on the two most predictive but contradictory cues to answer, they do not know which outcome to choose. We propose renaming this strategy the *hierarchical* strategy, based on additional pattern analyses described in the results section. (3) The *2* *vs. 1* strategy (M), in which participants base their responses on the presence or absence of the four cues but without attributing a probabilistic value, thus attributing each cue its absolute value (predicting chance performance for patterns 0101 and 1010). We propose renaming this the *equal weight* strategy. (4) The *one-cue* (*highly predictive;* G) or *single-cue strong* (M) strategy, in which participants attend selectively to one highly predictive cue (i.e., the presence or absence of a particular highly predictive cue) and ignore the other cues. When this cue is present, participants choose the outcome associated with it and when it is not present, participants choose the other outcome. (5) The *singleton + prototypes* strategy (M), in which participants learn how patterns with one single card (singleton) and patterns combining two congruent cues predict the outcome (i.e., 0001, 0010, 0011, 0100, 1000, and 1100). We propose renaming this the *congruent cues* strategy. (6) The *one-cue* (*less predictive;* G) or *single-cue weak* (M) strategy, in which participants attend selectively to one less predictive cue (i.e., the presence or absence of a particular less predictive cue) and ignore the other cues. When this cue is present, participants choose the outcome associated with it and when it is not present, participants choose the other outcome. (7) The *singleton* strategy (G and M), in which participants learn how the patterns containing only one cue predict the outcome (i.e., 0001, 0010, 0100, and 1000). (8) The *singleton strong* strategy (M), in which participants learn how the patterns containing only one highly predictive cue predict the outcome (i.e., patterns 0001 and 1000). (9) The *random* strategy (M), which is defined by the probability of random choices for all the patterns. We propose renaming this the *undetermined* strategy. Finally, we added another strategy that was not considered in previous studies: (10) the *two most predictive cues* strategy, in which participants attend selectively to the two highly predictive cues, whenever they are present. When the two most predictive but contradictory cues are present simultaneously, participants do not know which outcome to choose. Moreover, when these cues are not present participants do not learn how the patterns containing one less predictive cue predict the outcome. Thus, this strategy predicts chance performance for patterns 0010, 0100, 1011, and 1101.

**Table 2:** Descriptions of response strategies that may be used to solve the WPT.

**Strategies by author**			**Choices based on**
Gluck et al., 2002	Meeter et al., 2006	Bochud-Fragnière et al.	
Multicue	Perfect	Multicue	All combinations of cues
-	All but two strong cards	Hierarchical	Primarily on highly predictive cues Secondarily on less predictive cues Except in presence of both highly predictive cues
-	2 vs. 1	Equal weight	Combination of cues without hierarchy (i.e., without probabilistic value)
One-cue (highly predictive; 1000)	Single-cue strong (1000)	One-cue-1000 (highly predictive)	Highly predictive cue associated with sun
-	-	Two most predictive cues	Presence or absence of the two most predictive cues
One-cue (highly predictive; 0001)	Single-cue strong (0001)	One-cue-0001 (highly predictive)	Highly predictive cue associated with snow
-	Singleton + prototypes	Congruent cues	Patterns containing only one cue and their congruent combinations
One-cue (less predictive; 0010)	Single-cue weak (0010)	One-cue-0010 (less predictive)	Less predictive cue associated with snow
One-cue (less predictive; 0100)	Single-cue weak (0100)	One-cue-0100 (less predictive)	Less predictive cue associated with sun
Singleton	Singleton	Singleton	Patterns containing only one cue
-	Singleton strong	Singleton strong	Patterns containing only one of the most predictive cues
-	Random	Undetermined	Random or undetermined strategy

Strategies are listed from the most efficient (top) to the least efficient (bottom). As we modified the number of presentations of each pattern to fit the outcome probabilities and the total number of trials (i.e., 100), the two one-cue highly predictive strategies have slightly different maximal percentage of correct responses. The same is true for the two one-cue less predictive strategies.

Following the demonstration by Gluck et al. (2002) that strategies other than the multicue strategy may be used to solve the WPT tasks, we adopted their analysis methods and constructed ideal response profile models for each of the possible strategies (Table 2; Supplementary Table S1). We calculated these profiles by assuming that participants adhere strictly to this strategy throughout the entire task or for each block of 50 trials, respectively. However, it is essentially impossible for a participant to adhere strictly to a strategy across the entire task since participants obviously need at least a few trials to learn any rule and be able to reliably implement any given strategy, and they may also switch from one strategy to another during the task. Nevertheless, when the model predicted 100% correct responses for a particular pattern, participants should exhibit at least above chance performance. We compared the performance of each participant on each pattern with the performance predicted by each model in order to define the strategy that best fit the observed pattern of results for that participant. Mathematically, as described in Gluck et al. (2002), we summed the squared difference between the number of times outcome A (snow) was chosen by the participant and the number of times the outcome A (snow) was expected for each pattern. This sum was divided by the sum of squares of the number of presentations of each pattern to normalize the results. Therefore, the results could range from 0 to 1 for each model, with a lower score indicating a higher correspondence between the model and the participant’s response pattern. Gluck et al. (2002) also defined an arbitrary criterion to declare which particular strategy was used by a given participant. They reasoned that the strategy that obtained the lowest score below 0.1 fit the model best and was therefore the strategy used by the participant. Following previous studies (Gluck et al., 2002; Meeter et al., 2006; Mayor-Dubois et al., 2016), we analyzed the strategies for the entire 100 trials, as well as for the first block and the second block of 50 trials (i.e., trials 1–50 and trials 51–100).

We also compared statistically the scores of the different strategies for the group of participants who passed the task above chance level. We performed repeated-measures general linear model (GLM) analyses to compare the different means of the scores for each strategy. *Post-hoc* analyses were performed with paired-sample *t*-tests when F ratios were significant, thus controlling for Type I error rate (Carmer and Swanson, 1973). These analyses were carried out for the entire task (i.e., 100 trials), as well as for the first block and the second block of 50 trials separately.

To implement our novel pattern analyses and compare the performance, we first normalized the number of correct choices for each pattern by dividing it by the number of times it was presented. Note, however, that if our analyses were performed by pattern, participants had an equal number of chances to learn the influence of each individual cue (Table 1). We then performed two-tailed one-sample *t*-tests to determine whether participants performed better than chance for each pattern across the entire task. We did not perform these analyses for the two blocks of 50 trials separately since the number of presentations of certain patterns was too low for statistical analysis. We then took into consideration the congruence and the level of predictability of the cues and grouped together the following patterns: 0001 & 1000 (one highly predictive cue); 0010 & 0100 (one less predictive cue); 1100 & 0011 (two congruent cues); 0101 & 1010 (one highly predictive cue and one incongruent less predictive cue); 0111 & 1110 (two congruent cues and one incongruent less predictive cue); and 1011 & 1101 (two congruent cues and one incongruent highly predictive cue). The normalized number of correct choices for each category of patterns was compared with a repeated-measures general linear model (GLM) with *post-hoc* comparisons (paired samples *t*-test).

For the test trials, we used one-sample *t*-tests to determine whether participants could identify above chance which outcome each cue was associated with. We also performed a paired samples *t*-test to compare the participants’ performance with highly predictive cues vs. less predictive cues.

We did not use corrections for multiple comparisons for individual *t*-tests because in our study the risk of reporting a difference that may not exist (type I error) is not worse than the risk of missing a difference that may exist (type II error). Accordingly, we followed the recommendations of Rothman (1990), who argued that *“not making adjustments for multiple comparisons is preferable because it will lead to fewer errors of interpretation when the data under evaluation are not random numbers but actual observations on nature*,” and Saville (1990), who also argued that a procedure without correction is preferable because it provides greater consistency to compare results between studies. Although some comparisons might be more critical than others, considering all the comparisons is essential to characterize the behavior of participants. The most important indicator of the way participants considered each cue is the direction of the numerical difference for each individual comparison.

## Results

### Overall Performance

**Figure 2:**
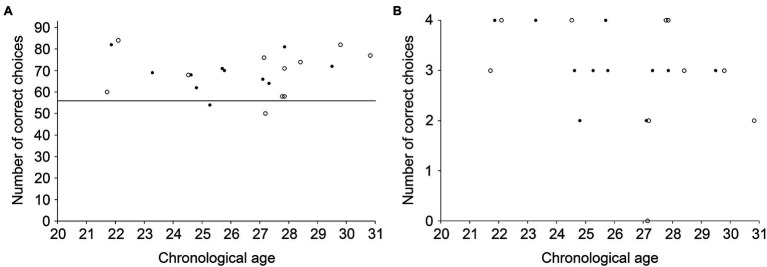
Individual performance of young healthy adults in the WPT (closed circles: women; open circles: men). **(A)** Number of correct choices across all 93 training trials. The black line represents the number of correct choices (56/93) defined as statistically different from chance at the individual level ($\chi_{\left(1\right)}^{2}$ = 3.882, *p* = 0.049). **(B)** Number of correct choices during the four test trials with individual cues and no feedback.

Our WPT comprised 100 trials, of which seven trials were excluded from our analyses since their patterns (0110, *n* = 3; 1001, *n* = 4) predicted both outcomes equally, and thus had no correct answer (see Table 1 for presentation of each combination of cues and their occurrences). As a group, participants made more correct choices than if they had chosen randomly across training trials (Figure 2A; *M* = 68.95, SD = 9.40; *t_(21)_* = 11.200, *p* < 0.001, *d_z_* = 2.388) and test trials (Figure 2B; *M* = 3.19, SD = 0.75, *t_(20)_* = 7.278, *p* < 0.001, *d_z_* = 1.588, one participant did not perform the test trials). Individually, 91 % of participants (20/22) performed above chance (≥ 56 correct choices; all $\chi_{\left(1\right)}^{2}$ ≥ 3.882, *p* ≤ 0.049). Only one 25-year-old female (54 correct choices) and one 27-year-old male (50 correct choices) failed the task. These two participants were thus excluded from subsequent analyses designed to characterize the learning strategies used to solve the WPT. The overall performance of the 20 participants who performed the training phase above chance level was at 76% of the perfect score across training trials (*M* = 70.65, SD = 8.00; above chance; *t_(19)_* = 13.497, *p* < 0.001, *d_z_* = 3.018) and at 81% of the perfect score for the four test trials (*M* = 3.26, SD = 0.73, *t_(18)_* = 7.507, *p* < 0.001, *d_z_* = 1.722).

### Modeled Strategies

Following the analysis method defined by Gluck et al. (2002), we first determined for each participant how many modeled strategies obtained a score < 0.1, and could therefore be considered a strategy likely used to solve the task (Supplementary Tables S2.1–S2.3). The first analysis including all training trials revealed an average of 6.50 strategies with a score < 0.1 per participant (range: 2–10; SD = 1.96). Considering only the first 50 trials, we found an average of 4.85 strategies with a score < 0.1 per participant (range: 1–9; SD = 2.56). Considering only the last 50 trials, we found an average of 4.35 strategies with a score < 0.1 per participant (range: 0–7; SD = 2.46). Several strategies were thus below the cutoff score of 0.1 to be considered as likely strategies based on Gluck et al.’s criterion. However, this method cannot be used to attribute a specific strategy to each participant, because the fit of different strategies cannot be compared statistically at the individual level.

**Figure 3:**
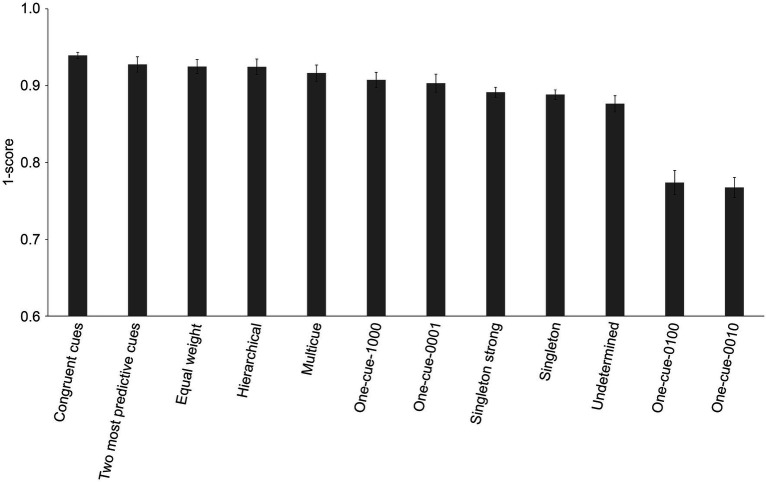
Means of fitted scores (±SE) for each strategy for the group of 20 young adults who performed the WPT above chance level. Note that in order to make the graphical representation more intuitive, the y-axis represents 1-score generated by the model. Therefore, the higher the value, the more likely it was that a given strategy was used by the group of participants. The strategies are listed in ranking order from the most likely used (congruent cues) to the least likely used (one less predictive cue).

We therefore performed a statistical comparison of the different strategies that may have been used to solve the WPT at the group level, in order to determine which strategy was most likely used by our group of healthy young adults. Over the entire block of training trials, different strategies obtained different scores (Figure 3; Supplementary Table S2.1; *F*_(11,209)_ = 37.637, *p* < 0.001, $\eta_{p}^{2}$ = 0.665). *Post-hoc* comparisons revealed differences between the congruent cues strategy (*M* = 0.0607; SD = 0.0168) and the following strategies: multicue (*M* = 0.0837; SD = 0.0439), one-cue-1000 (*M* = 0.0926; SD = 0.0408), one-cue-0001 (*M* = 0.0967; SD = 0.0483), singleton strong (*M* = 0.1086; SD = 0.0271), singleton (*M* = 0.1117; SD = 0.0258), undetermined (*M* = 0.1235; SD = 0.0433), one-cue-0100 (*M* = 0.2261; SD = 0.0653) and one-cue-0010 (*M* = 0.2325; SD = 0.0539; all *p* < 0.05). However, there were no statistically significant differences between the congruent cues strategy and the two most predictive cues (*M* = 0.0724; SD = 0.0414), equal weight (*M* = 0.0751; SD = 0.0375) or hierarchical (*M* = 0.0755; SD = 0.0417) strategies.

The same analysis performed on the first block of 50 training trials also revealed differences in the scores for different strategies (Supplementary Figure S2; Supplementary Table S2.2; *F*_(11,209)_ = 17.046, *p* < 0.001, $\eta_{p}^{2}$ = 0.473). *Post-hoc* comparisons revealed differences between the congruent cues strategy (*M* = 0.0769; SD = 0.0410) and the following strategies: equal weight (*M* = 0.0933; SD = 0.0493), hierarchical (*M* = 0.1002; SD = 0.0486), multicue (*M* = 0.1087; SD = 0.0530), one-cue-1000 (*M* = 0.1146; SD = 0.0500), one-cue-0001 (*M* = 0.1216; SD = 0.0581), singleton strong (*M* = 0.1068; SD = 0.0457), singleton (*M* = 0.1107; SD = 0.0516), undetermined (*M* = 0.1132; SD = 0.0562), one-cue-0100 (*M* = 0.2193; SD = 0.0830) and one-cue-0010 (*M* = 0.2447; SD = 0.1078; all *p* < 0.05), but not with the two most predictive cues strategy (*M* = 0.0964; SD = 0.0473).

The same analysis performed on the second block of 50 training trials similarly revealed differences in the scores for different strategies (Supplementary Figure S2; Supplementary Table S2.3; *F*_(11,209)_ = 23.182, *p* < 0.001, $\eta_{p}^{2}$ = 0.550). *Post-hoc* comparisons revealed differences between the congruent cues strategy (*M* = 0.0817; SD = 0.0321) and the following strategies: one-cue-1000 (*M* = 0.1196; SD = 0.0572), singleton strong (*M* = 0.1436; SD = 0.0352), singleton (*M* = 0.1455; SD = 0.0266), undetermined (*M* = 0.1650; SD = 0.0425), one-cue-0010 (*M* = 0.2648; SD = 0.0895), one-cue-0100 (*M* = 0.2650; SD = 0.0947; all *p* < 0.001). In contrast, there were no statistically significant differences between the congruent cues strategy and the following strategies: two most predictive cues (*M* = 0.0891; SD = 0.0629), hierarchical (*M* = 0.0910; SD = 0.0615), equal weight (*M* = 0.0975; SD = 0.0561), multicue (*M* = 0.1005; SD = 0.0664) and one-cue-0001 (*M* = 0.1155; SD = 0.0739).

In sum, considering all 100 training trials, the first block of 50 trials or the last block of 50 trials, the modeled strategies analysis suggested that the congruent cues strategy was the most likely used by this group of young healthy adults who performed above chance level. However, specific statistical comparisons did not always enable the distinction between different modeled strategies, especially between the congruent cues, the two most predictive cues, the equal weight, and the hierarchical strategies. Thus, in order to identify the strategy used by the majority of participants, it was necessary to analyze their responses to individual patterns of cues, for which the different learning strategies lead to different predictions.

### Individual Patterns

We analyzed the performance of our group of successful participants for each pattern of cues. This resulted in a performance profile that could be compared to the theoretical profile illustrating the maximal percentage of correct responses possible for each pattern of cues when using a given learning strategy (Supplementary Table S1). Therefore, if an observed profile corresponded to a theoretical profile, this would suggest that this specific strategy was used.

Considering all training trials (Table 3), the overall group performance was above chance for all the patterns except for the two patterns that included the two highly predictive cues and one of the two less predictive cues (patterns 1011 and 1101). The fact that, as a group, our participants did not score above chance with patterns 1011 and 1101 indicates that they did not use the multicue strategy to solve the task since use of the multicue strategy should enable above chance performance for all cue combinations, including these two patterns. In contrast, these results are consistent with the use of the hierarchical strategy, which enables above chance performance for all patterns (i.e., 0001, 0010, 0011, 0100, 0101, 0111, 1000, 1010, 1100, and 1110), except when the two most predictive cues are present simultaneously (i.e., 1011 and 1101; Supplementary Tables S1, S3).

**Table 3:** Two-tailed *t*-tests comparing the performance of the group of participants with chance level for each individual pattern.

Patterns	100 trials					
	Mean	SD	*n*	*t_(19)_*	*p*	*Cohen’s d_z_*
0001	7.20	1.67	9	7.216	<0.001^**^	1.614
0010	3.30	1.66	5	2.158	0.044^**^	0.483
0011	11.20	1.40	13	15.022	<0.001^**^	3.359
0100	2.90	1.12	4	3.596	0.002^**^	0.804
0101	3.95	1.54	6	2.762	0.012^**^	0.618
0110^a^	–	–	3	–	–	–
0111	6.70	2.11	10	3.611	0.002^**^	0.808
1000	7.70	1.38	9	10.368	<0.001^**^	2.318
1001^a^	–	–	4	–	–	–
1010	4.70	1.59	7	3.369	0.003^**^	0.753
1011	1.55	0.89	3	0.252	0.804	0.056
1100	11.60	1.31	13	17.359	<0.001^**^	3.882
1101	2.80	1.24	5	1.082	0.293	0.242
1110	7.05	1.50	9	7.585	<0.001^**^	1.696

a The patterns including either the two highly predictive cues (1001) or the two less predictive cues (0110) do not have a correct answer but are nonetheless listed for the sake of completeness.

** Indicate *p* < 0.05 for two-tailed one-sample *t*-test comparisons. *n* represents the number of presentations of each pattern.

### Categories of Patterns

**Figure 4:**
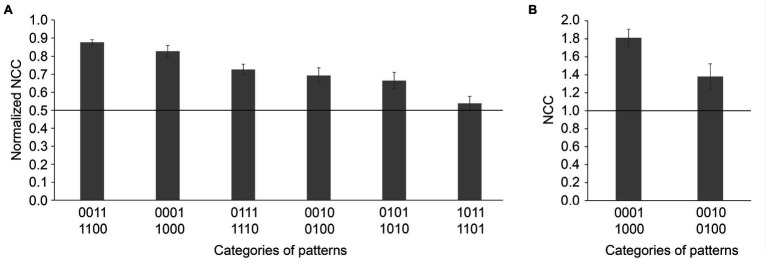
**(A)** Normalized number of correct choices (NCC; mean ± SE) during the training phase for each category of patterns for the participants who performed the WPT above chance level; **(B)** Number of correct choices (NCC; mean ± SE) during the test phase for each category of patterns for the participants who performed the WPT above chance level.

The previous analysis revealed how well participants performed for each pattern of cues, but not how the different categories of cues (highly vs. less predictive) may have influenced their choices. We therefore performed an analysis of the different groups of patterns based on their congruence and level of predictability. Considering the entire block of 100 training trials, we found differences in the normalized number of correct choices for the different categories of patterns (Figure 4A; *F*_(5,95)_ = 14.472, *p* < 0.001, $\eta_{p}^{2}$ = 0.432). Group performance was above chance for all categories of patterns except when the two highly predictive cues were present simultaneously: 0011 & 1100 (two congruent cues; *M* = 0.88, SD = 0.07, t_(19)_ = 25.259, *p* < 0.001, *d_z_* = 5.648), 0001 & 1000 (one highly predictive cue; *M* = 0.83, SD = 0.14, *t*_(19)_ = 10.174, *p* < 0.001, *d_z_* = 2.275), 0111 & 1110 (two congruent cues and one incongruent less predictive cue; *M* = 0.73, SD = 0.13, *t*_(19)_ = 7.650, *p* < 0.001, *d_z_* = 1.711), 0010 & 0100 (one less predictive cue; *M* = 0.69, SD = 0.19, *t*_(19)_ = 4.457, *p* < 0.001, *d_z_* = 0.997), 0101 & 1010 (one highly predictive cue and one incongruent less predictive cue; *M* = 0.66, SD = 0.20, *t*_(19)_ = 3.638, *p* = 0.002, *d_z_* = 0.813), 1011 & 1101 (two congruent cues and one incongruent highly predictive cue; *M* = 0.54, SD = 0.17, *t*_(19)_ = 1.010, *p* = 0.325, *d_z_* = 0.226; *NB*: recall that we did not analyze patterns 1001 & 0110 since there was no correct response).

Importantly, *post-hoc* analyses revealed that the participants who passed the task above chance had a higher normalized number of correct choices for the category that included patterns 0011 & 1100 than the categories that included patterns 0111 & 1110, 0010 & 0100, 0101 & 1010, and 1011 & 1101 (all *p* < 0.001), suggesting that congruent less predictive cues acted synergistically and that incongruent less predictive cues acted antagonistically (Table 4). However, the presence of a less predictive cue congruent with a highly predictive cue did not improve performance, as compared to when only one highly predictive cue was present (0011 & 1100 vs. 0001 & 1000, *p* = 0.106), likely due to a ceiling effect. The predominant influence of the two highly predictive cues was further supported by the findings that participants exhibited a higher normalized number of correct choices for patterns 0001 & 1000 than for patterns 0111 & 1110, 0101 & 1010, 1011 & 1101, 0010 & 0100 (all *p* < 0.05), and a higher normalized number of correct choices for 0111 & 1110 than 1011 & 1101, and for 0010 & 0100 than 1011 & 1101 (both *p* < 0.05). Although participants had a higher normalized number of correct choices for 0101 & 1010 than 1011 & 1101, this comparison failed to reach the predefined level of statistical significance (*p* = 0.059, two-tailed).

**Table 4:** *p* values and Cohen’s *d*_z_ for the *post-hoc* paired *t*-test comparisons between the different categories of patterns.

	Values	0011 & 1100	0001 & 1000	0111 & 1110	0010 & 0100	0101 & 1010	1011 & 1101
0011 & 1100	*p*	–					
	*d_z_*	–					
0001 & 1000	*p*	0.106	–				
	*d_z_*	0.380	–				
0111 & 1110	*p*	<0.001^**^	<0.001^**^	–			
	*d_z_*	1.451	0.921	–			
0010 & 0100	*p*	<0.001^**^	0.026^**^	0.498	–		
	*d_z_*	0.993	0.539	0.154	–		
0101 & 1010	*p*	<0.001^**^	<0.001^**^	0.222	0.685	–	
	*d_z_*	1.036	0.905	0.282	0.092	–	
1011 & 1101	*p*	<0.001^**^	<0.001^**^	<0.001^**^	0.004^**^	0.059^*^	–
	*d_z_*	2.263	1.496	1.194	0.736	0.449	–

* indicates *p* values <0.10 for two-tailed paired *t*-test comparisons, which would be equivalent to *p* values <0.05 for one-tailed comparisons.

** Indicate *p* < 0.05 for two-tailed paired *t*-test comparisons.

In accordance with training trials analyses, overall group performance on the test trials was above chance for the two categories of patterns: 0001 & 1000 (*M* = 1.84, *SD* = 0.38, *t_(18)_* = 9.798, *p* < 0.001, *d_z_* = 2.248) and 0010 & 0100 (*M* = 1.42, *SD* = 0.61, *t_(18)_* = 3.024, *p* = 0.007, *d_z_* = 0.694), confirming that participants knew the outcomes associated with the two highly predictive cues (0001 & 1000) and the two less predictive cues (0010 & 0100). Moreover, a comparison of the participants’ performance during the test trials on these two different categories of patterns showed that participants had a higher performance for highly predictive cues than for less predictive cues (Figure 4B; *t_(18)_* = 2.650, *p* = 0.016, *d_z_* = 0.608).

Altogether, these data show that participants learned the relationships between individuals cues and outcomes, for both highly and less predictive cues. However, the influence of the two less predictive cues was weaker than is predicted by the multicue strategy. The two less predictive cues influenced performance when only one or no highly predictive cue was present, but not when the two highly predictive cues associated with opposite outcomes were present simultaneously. This pattern of results is thus more consistent with the hierarchical strategy: participants based their choices primarily on the presence or absence of the highly predictive cues and considered the two less predictive cues secondarily.

## Discussion

The aim of the current study was to characterize the learning strategies and cognitive processes used to solve the Weather Prediction Task (WPT). We identified an additional learning strategy and developed a new way of analyzing the data that can exclude alternative strategies and thus better characterize the cognitive processes enabling above chance performance in the WPT. Consistent with previous results (Gluck et al., 2002; Meeter et al., 2006), we found that the majority of young adult participants did not solve the WPT using a multicue strategy as presumed by Knowlton et al. (1994). As a group, our participants learned the correct outcome for each individual cue, but their level of performance was higher for the highly predictive cues than for the less predictive cues. Moreover, although participants learned the correct outcomes associated with the less predictive cues, the presence of one of these cues did not influence the participants’ choices when the two highly predictive cues were also present simultaneously (i.e., patterns 1011 and 1101, patterns in which the outcome associations of the two less predictive cues are necessary to determine the correct outcome). The participants’ choices were thus consistent with the hierarchical strategy: The highly predictive cues have a greater influence on the participants’ choices than the less predictive cues; participants consider the highly predictive cues primarily and the less predictive cues secondarily only when the two highly predictive cues are not present simultaneously. This strategy may reflect a conditional learning process dependent on the hippocampal formation which suggests, in agreement with previous studies (Knowlton et al., 1996a; Poldrack et al., 2001; Gluck et al., 2002; Hopkins et al., 2004; Foerde et al., 2006, 2007; Lagnado et al., 2006; Meeter et al., 2006; Newell et al., 2007; Price, 2009; Li et al., 2016), that the WPT does not primarily assess non-declarative learning and memory processes.

### Comparison With Previous Studies

In agreement with previous results (Gluck et al., 2002; Meeter et al., 2006), we found that most young healthy adults did not use the multicue strategy. Moreover, participants may use different learning strategies that can lead to similar overall performance in the WPT. To determine which strategy any given participant may have used, Gluck et al. (2002) proposed a specific method to analyze WPT data. They compared the responses of each participant to ideal response profiles generated for each learning strategy and reasoned that the strategy obtaining the lowest score below an arbitrary criterion was used by the participant, because it fit their performance best. In their study, they only took into consideration the four strategies that participants reported to have used. This method, when used to analyze these four potential strategies, enabled the authors to obtain only one score that was under the criterion for the majority of participants. However, as discussed by Meeter et al. (2006), identifying all possible strategies is critical, as participants will be improperly classified if the strategy that they used is not included in the analysis. All possible strategies must therefore be identified *a priori* and included in the strategy analysis. In the current study, we not only identified another strategy, the *two most predictive cues* strategy, which had been previously overlooked, but we also found that for the majority of our participants several strategies reached the performance criterion. Indeed, since very few trials can be used to distinguish the different strategies, statistically indistinguishable fitted scores were generated for several strategies for a majority of participants. This implies that more than one learning strategy could be attributed to most successful participants.

We therefore performed statistical comparisons of potentially used strategies at the group level, which identified the congruent cues strategy as being the strategy with the highest probability of being used by successful participants. However, the average score of this strategy did not differ from the equal weight, the two most predictive cues, and the hierarchical strategies. Note also that the average performance reached by successful participants (76% of the optimal score) was more closely aligned with performance that would be obtained when using the congruent cues strategy (78.5%) than with any other strategy. However, one cannot use global performance as an indicator of the strategy used because strategy analyses are based on the participants’ theoretical responses (i.e., perfectly adhering to a single strategy across all trials) without taking into consideration the learning phase, errors, guesses, strategy changes, as well as strategy and performance differences between participants. At the individual level, it is obvious, for example, that no participant can adhere perfectly to a strategy from trial 1 to trial 100 (or not likely even from trial 51 to 100). At the group level, if half of the participants performed at 60% and half performed at 90%, the overall performance will be 75%, even if no participant obtained a score of 75%. Thus, although traditional strategy analyses suggested participants were using the congruent cues strategy, this finding was unreliable.

Previous studies evaluating the learning strategies used by healthy participants during the WPT or the Ice Cream Task usually exposed participants to 200 trials (Gluck et al., 2002; Meeter et al., 2006; Mayor-Dubois et al., 2016). However, because we designed our task to be used with pre-school aged children, we limited training to 100 trials. In order to ensure that shortening the task did not influence the results of our strategy analyses, we replicated our study and tested an additional 15 young healthy adults on the same task with 200 trials (Supplementary Material). We did not find any significant differences in performance, learning strategy and pattern analyses between the 100-trial-WPT, and the first 100 trials of our 200-trial-WPT, thus confirming the findings of the first 100 trial experiment. Moreover, in accordance with the 100-trial-WPT, participants did not respond correctly for all the patterns of cues even with twice as many presentations of each cue or individual pattern, in particular patterns 1011 and 1101. Importantly, although the number of presentations of patterns 1011 & 1101 (*n* = 16) in the 200-trial-WPT was still inferior to some patterns in the 100-trial-WPT, this number was superior to the number of presentations of the following patterns: 0010 & 0100 (*n* = 9), 0101 & 1010 (*n* = 13) for which participants responded correctly in the 100-trial-WPT. Thus, despite an increased number of presentations in the 200-trial-WPT, participants only showed a tendency to respond correctly for pattern 1011 (*n* = 6) but not for pattern 1101 (*n* = 10). Similar to the 100-trial-WPT, participants showed the ability to learn the correct outcome for each individual cue, and their level of performance was also numerically higher for the highly predictive cues than the less predictive cues. Although participants learned the correct outcome for the less predictive cues alone, they did not answer correctly when the two highly predictive cues were presented simultaneously (i.e., patterns 1011 and 1101; when the two less predictive cues were necessary to determine the correct outcome). Interestingly, although pattern analyses performed on the entire 200-trial-WPT may suggest an increasing influence of the two less predictive cues on the participants’ responses, their responses remained consistent with the use of the hierarchical strategy. Thus, the fundamental information about the cognitive processes implicated in the resolution of the WPT can already be extracted from performance on tasks with only 100 trials.

### Pattern Analyses: Necessary and Sufficient

In contrast to previous modeled strategy analyses (Gluck et al., 2002; Meeter et al., 2006), our pattern analyses revealed a profile of performance that perfectly matched the hierarchical strategy (better than chance performance on all patterns except 1011 and 1101, a strategy referred to as the *all but two strong cards* strategy in Meeter et al. (2006)). Moreover, comparing the results of our pattern analyses to the maximal percentage of correct responses for each pattern excluded the use of all other strategies by the majority of our participants. Specifically, the performance of our young healthy participants was not consistent with the *multicue* strategy, or else they should have had better than chance performance on all the individual patterns, including patterns 1011 and 1101. The performance of our participants was not consistent with the *equal weight* strategy, or else they should have had better than chance performance on patterns 1011 and 1101 and at chance performance on patterns 0101 and 1010. The performance of our participants was not consistent with either one of the *one-cue highly predictive* strategies (i.e., one-cue-1000 and one-cue-0001), or else they should have had better than chance performance on pattern 1101 or pattern 1011, respectively, and lower than chance performance on patterns 0100 and 1011 or patterns 0010 and 1101, respectively. Most importantly, and in contrast to what the modeled strategy analyses suggested, the performance of our participants was not consistent with the *two most predictive cues* nor the *congruent cues* strategies, or else their performance should have been at chance for patterns 0010 and 0100 for the two most predictive cues strategy, or at chance for patterns 0101, 0111, 1010 and 1110 for the congruent cues strategy. Our participants’ performance was not consistent with the *one-cue less predictive* strategy, or else their performance should have been better than chance for patterns 1011 and 1101 and lower than chance for patterns 0001, 0101, 1010, and 1110 (for one-cue-0010) and lower than chance for patterns 0101, 0111, 1000, and 1010 (for one-cue-0100). Finally, our participants’ performance was not consistent with the *singleton*, *singleton strong* and the *undetermined* strategies, which predict chance performance for a majority of the individual patterns. Nonetheless, we cannot exclude the fact that a *minority* of participants used other strategies to solve the WPT than the one defined at the group level. Moreover, since some patterns were not present in sufficient number or at all in some windows, we could not perform pattern analyses on blocks of 25 trials (or even 50 trials) in order to identify possible switches between different strategies (Gluck et al., 2002; Meeter et al., 2006).

In sum, both the strategy and pattern analyses demonstrated that the hierarchical strategy was likely employed by most participants to solve the WPT. However, whereas traditional strategy analyses identified several potential but statistically indistinguishable strategies, pattern analyses demonstrated that only the hierarchical strategy corresponded to the participants’ actual performance across all training trials. Finally, and most importantly, pattern analyses do not require *a priori* identification of all potentially used strategies. Altogether, our findings lead us to conclude that modeled strategy analyses are not useful and that only pattern analyses need be performed on data collected using the WPT.

### A Hierarchical Processing of Cues

If pattern analyses could only be used to illustrate the profile of performance of our participants across all patterns, we could have retained the nomenclature of Meeter et al. (2006): the *all but two strong cards* strategy. However, whereas Meeter et al. (2006) used this term to describe the strategy that was consistent with the global performance of participants, our pattern analyses provided evidence regarding the cognitive processes engaged in solving the WPT. Specifically, by considering every pattern, our analyses were able to demonstrate how participants processed each individual cue in a particular pattern. Our analyses showed that participants learned the correct outcome for all patterns except 1011 & 1101 (i.e., patterns containing the two highly predictive cues and one less predictive cue). Although this suggests that our participants did not learn the outcome associated with the two less predictive cues, their performance on other training trials and test trials when the cues were presented alone showed that they knew which outcome was associated with each of the four cues. Nonetheless, when taken together, superior performance for highly predictive cues than for less predictive cues during both training and test trials, and the lack of a significant impact of the less predictive cues in the presence of the two highly predictive cues suggests that participants were treating the cues in a hierarchical manner.

Reliance on a hierarchical strategy is supported by the comparison of the performance with different patterns during training, which showed that both the highly predictive cues and the less predictive cues had an impact on the participants’ choices. However, the impact of the highly predictive cues was stronger than that of the less predictive cues. First, there was a beneficial effect of a congruent highly predictive cue since our participants performed better on 0011 & 1100 than on 0010 & 0100. In contrast, there was a detrimental effect of an incongruent highly predictive cue since our participants performed better on 0011 & 1100 than on 1011 & 1101. Second, there was a detrimental effect of an incongruent less predictive cue since our participants performed better on 0001 & 1000 than on 0101 & 1010, and better on 0011 & 1100 than on 0111 & 1110. In contrast, our data also suggest a beneficial effect of a congruent less predictive cue, because participants performed better on 0011 & 1100 than on 0001 & 1000, although the difference was only tendential likely due to a ceiling effect. Third, we showed that a highly predictive cue had a stronger impact than a less predictive cue on participants’ choices as they performed better on 0111 & 1110 than on 1011 & 1101. Nonetheless, the less predictive cues were not simple distractors, but rather cues taken into consideration when determining the participants’ choices, as evidenced by the fact that participants performed better on 0001 & 1000 and 0011 & 1100 than on 0101 & 1010.

Whereas all of the above-mentioned analyses demonstrated that participants paid attention to all four cues when responding, they also further support the hypothesis that participants employed a hierarchical strategy. Indeed, participants primarily based their responses on the presence or absence of the two highly predictive cues, and considered the two less predictive cues secondarily, except when both highly predictive cues were present simultaneously. Although less predictive cues have an impact on the participants’ choices, they have less of an impact than would be expected if participants were using a multicue strategy. Importantly, whereas our data suggest a hierarchical treatment of the cues, neither our experimental design nor our analyses allow us to determine whether this hierarchical treatment of information follows a sequential or temporal organization with highly predictive cues being considered first, and less predictive cues being considered second. The hierarchical strategy described here is more consistent with a flexible learning and memory process than with a stimulus response or procedural (habit) learning and memory process.

Finally, it is important to note that the entire corpus of results including all comparisons described in the results section converge to draw this conclusion and that no single result leads to an alternative explanation. Even if one considers the few comparisons for which the value of *p* does not reach the predefined level of statistical significance, the observed pattern of results remains consistent with the use of a hierarchical strategy. Indeed, the use of a hierarchical strategy is primarily suggested by the fact that (1) participants know the correct outcome for each individual cue, but not when one less predictive cue is combined with the two highly predictive cue (i.e., patterns 1011 and 1101); (2) performance is higher for the highly predictive cues than the less predictive cues alone, during training and test trials; (3) the comparisons between the different categories of patterns indicate that both the less predictive cues and the highly predictive cues have an impact on the participants’ responses, but that the less predictive cues have a lower impact than the highly predictive cues. Moreover, although we normalized the number of correct choices for each pattern, participants had an equal number of chances to learn the influence of each individual cue, and our analyses reflected how participants processed each cue. Our analyses also clearly revealed that participants did not learn each pattern independently and that the number of presentations of each pattern did not impact the results; otherwise, it would be difficult to explain why participants performed above chance with patterns 0010 & 0100, but not with patterns 1011 & 1101. The replication of these findings in our experiment with 200 trials further supports this interpretation.

## Conclusion

We found that, as a group, healthy young adults did not use the multicue strategy to solve the WPT as was initially proposed (Knowlton et al., 1994). Instead, our data show that they used what we describe as the hierarchical strategy: participants processed the highly predictive cues primarily and the less predictive cues secondarily, but only when the two highly predictive cues were not present simultaneously. This hierarchical strategy has not been considered as such by previous authors. Our findings highlight the importance of clearly identifying the cognitive processes that may be engaged when solving a memory task. As previously noted by Gluck et al. (2002), our findings further emphasize that the results of a global analysis of a complex cognitive task (e.g., the overall number of correct choices) are rarely sufficient to identify the cognitive processes implicated in its resolution. Our findings also support previous studies showing that the WPT does not primarily assess non-declarative learning and memory processes. Accordingly, the results of previous studies should be revisited in light of the present findings and, most importantly, the WPT should not be considered as a pure task to assess whether a particular population exhibit striatal or non-declarative memory dysfunction.

## Data Availability Statement

The raw data supporting the conclusions of this article will be made available by the authors, without undue reservation.

## Ethics Statement

The studies involving human participants were reviewed and approved by the Cantonal Ethics Commission for Human Research [Vaud, Switzerland; project no. PB_2017-00074 (60/14)]. The participants provided their written informed consent to participate in this study.

## Author Contributions

PBL, EB-F, and PL were responsible for the conception and design of the work and interpretation of the data. EB-F was responsible for data acquisition and wrote the first draft of the manuscript. EB-F and PL were responsible for data analysis. PBL and PL were responsible for the acquisition of funding and the supervision of the work. All authors have critically revised the manuscript for publication and agreed to be accountable for all aspects of the work. All authors contributed to the article and approved the submitted version.

## Funding

This research was supported by grant 100019_165481 from the Swiss National Science Foundation to PL and PBL and the Faculty of Social and Political Sciences of the University of Lausanne.

## Conflict of Interest

The authors declare that the research was conducted in the absence of any commercial or financial relationships that could be construed as a potential conflict of interest.

## Publisher’s Note

All claims expressed in this article are solely those of the authors and do not necessarily represent those of their affiliated organizations, or those of the publisher, the editors and the reviewers. Any product that may be evaluated in this article, or claim that may be made by its manufacturer, is not guaranteed or endorsed by the publisher.
