# Supplementary material for: What Is the Weather Prediction Task Good for? A New Analysis of Learning Strategies Reveals How Young Adults Solve the Task
Source: Front Psychol. 2022 Jun 13;13:886339. doi: 10.3389/fpsyg.2022.886339 (PMC9234396; doi:10.3389/fpsyg.2022.886339)
Supplement: Supplementary file 1 [file Data_Sheet_1.docx]

**Figure S1.** Representation of the four sets of cues (sea animals) that were used for the modified WPT. Each cue was randomly assigned one outcome probability, and that cue-outcome association probability remained fixed for all participants who were tested with that set of cues. Since there was no consistent difference^[[1]](#footnote-1)^ in performance between sets (data not shown), data obtained with all four sets were combined for the presentation of the results in the main part of the manuscript.


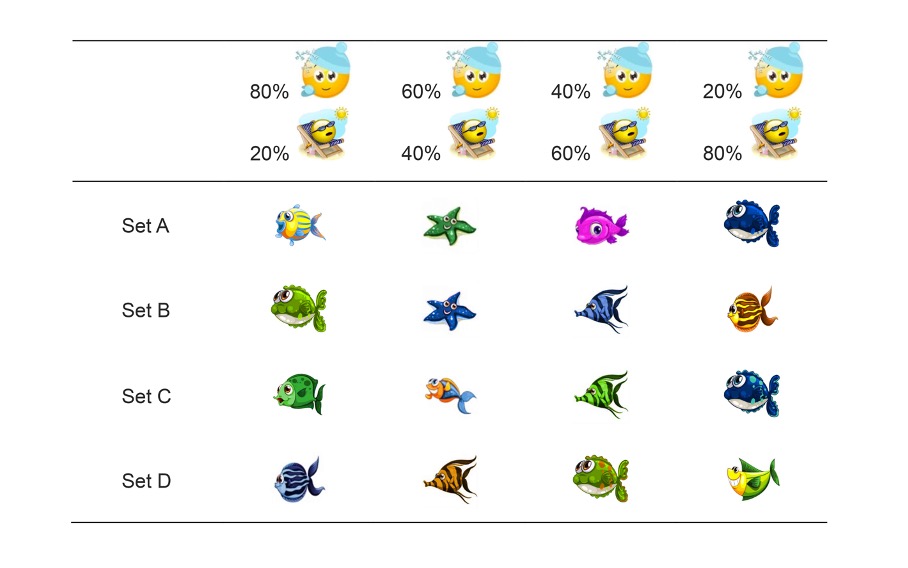


**Figure S2.** Means of fitted scores (± SE) on the first and last blocks of 50 trials for each strategy for the group of 20 young adults who performed the WPT above chance level. Note that in order to make the graphical representation more intuitive, the y axis represents 1-score generated by the model. Therefore, the higher the value, the more likely was a given strategy to be used by the group of participants. To simplify comparisons, strategies are listed in ranking order from the most likely used across the 100 trials (congruent cues) to the least likely used (one less predictive cue). Importantly, note that the mean of the fitted scores for each strategy across the 100 trials (**Figure 3**) does not correspond to a mathematical mean of the scores of the two blocks of 50 trials presented here.


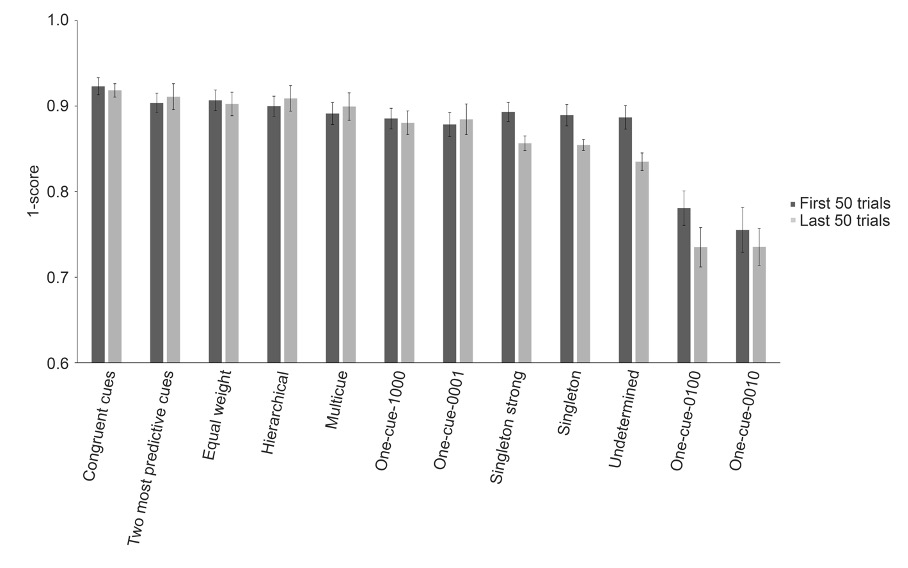


**Statistical comparison of the strategies used in the two blocks of trials.**

There were differences in the scores for different strategies (F_(11,209)_ = 33.047, p < .001, η^2^_p_ = .635) and a significant interaction between strategies and blocks of trials (F_(11,209)_ = 1.877, p = .044, η^2^_p_ = .090), but no statistically significant differences between the two blocks of training trials (F_(1,19)_ = 3.069, p = .096, η^2^_p_ = .139). See Main Text for detailed description of the analyses across the 100 trials and the two blocks of 50 trials.

1. When performing the strategy analyses across the first block of 50 trials (section 3.2), we found a spurious interaction effect between strategy and set (*F*_(33,132)_ = 1.595, *p* = .034, η2p = .285) on the score of the strategies. In all other analyses, the effect of sets was not significant (data not shown). [↑](#footnote-ref-1)
