# Supplementary material for: What Is the Weather Prediction Task Good for? A New Analysis of Learning Strategies Reveals How Young Adults Solve the Task
Source: Front Psychol. 2022 Jun 13;13:886339. doi: 10.3389/fpsyg.2022.886339 (PMC9234396; doi:10.3389/fpsyg.2022.886339)
Supplement: Supplementary file 2 [file Data_Sheet_2.docx]

| **Table S1.** *Response strategies, percentage of optimal responses across the entire task and maximal percentage of correct responses for each pattern.* |
| --- |

| **Strategies** | **%** | **Patterns** | | | | | | | | | | | | | |
| --- | --- | --- | --- | --- | --- | --- | --- | --- | --- | --- | --- | --- | --- | --- | --- |
|  |  | **0001** | **0010** | **0011** | **0100** | **0101** | **0110** | **0111** | **1000** | **1001** | **1010** | **1011** | **1100** | **1101** | **1110** |
|  |  | ***n* = 9** | ***n* = 5** | ***n* = 13** | ***n* = 4** | ***n* = 6** | ***n* = 3** | ***n* = 10** | ***n* = 9** | ***n* = 4** | ***n* = 7** | ***n* = 3** | ***n* = 13** | ***n* =5** | ***n* = 9** |
| **Multicue** | 100% | 100% | 100% | 100% | 100% | 100% | 100% | 100% | 100% | 100% | 100% | 100% | 100% | 100% | 100% |
| **Hierarchical** | 95.7% | 100% | 100% | 100% | 100% | 100% | 100% | 100% | 100% | 100% | 100% | 50% | 100% | 50% | 100% |
| **Equal weight** | 93% | 100% | 100% | 100% | 100% | 50% | 100% | 100% | 100% | 100% | 50% | 100% | 100% | 100% | 100% |
| **One-cue-1000 (highly predictive)** | 92.5% | 100% | 100% | 100% | 0% | 100% | 100% | 100% | 100% | 100% | 100% | 0% | 100% | 100% | 100% |
| **Two most predictive cues** | 90.9% | 100% | 50% | 100% | 50% | 100% | 100% | 100% | 100% | 100% | 100% | 50% | 100% | 50% | 100% |
| **One-cue-0001 (highly predictive)** | 89.2% | 100% | 0% | 100% | 100% | 100% | 100% | 100% | 100% | 100% | 100% | 100% | 100% | 0% | 100% |
| **Congruent cues** | 78.5% | 100% | 100% | 100% | 100% | 50% | 100% | 50% | 100% | 100% | 50% | 50% | 100% | 50% | 50% |
| **One-cue-0010 (less predictive)** | 66.7% | 0% | 100% | 100% | 100% | 0% | 100% | 100% | 100% | 100% | 0% | 100% | 100% | 100% | 0% |
| **One-cue-0100 (less predictive)** | 65.6% | 100% | 100% | 100% | 100% | 0% | 100% | 0% | 0% | 100% | 0% | 100% | 100% | 100% | 100% |
| **Singleton** | 64.5% | 100% | 100% | 50% | 100% | 50% | 100% | 50% | 100% | 100% | 50% | 50% | 50% | 50% | 50% |
| **Singleton strong** | 59.7% | 100% | 50% | 50% | 50% | 50% | 100% | 50% | 100% | 100% | 50% | 50% | 50% | 50% | 50% |
| **Undetermined** | 50% | 50% | 50% | 50% | 50% | 50% | 100% | 50% | 50% | 100% | 50% | 50% | 50% | 50% | 50% |
| The maximal percentage of correct responses for each pattern are based on the answers that should be given by a participant following the strategy. For example, a participant following the one-cue-1000 strategy will answer outcome B (sun) in presence of the cue number 1000 (i.e., 1000, 1001, 1010, 1011, 1100, 1101, and 1110), but outcome A (snow) in its absence (i.e., 0001, 0010, 0011, 0100, 0101, 0110, and 0111). It is essentially impossible for a participant to adhere strictly to a strategy across the entire task since participants obviously need at least a few trials to learn any rule and be able to reliably implement any given strategy, and they may also switch from one strategy to another during the task. Nevertheless, when the model predicted 100% correct responses for a particular pattern, participants should exhibit at least above chance performance. In contrast, when the model predicted 0% correct responses for a particular pattern, participants should exhibit a performance which is below chance level. | | | | | | | | | | | | | | | |

**Table S2.1.** *Scores (actual response - theoretical response; Gluck et al., 2002) for all the strategies for each participant who performed the task above chance level across the 100 training trials. Number of strategies with a score inferior to 0.1, the criterion considered by Gluck et al (2002) to select possible strategies used to solve the task.*

|  | **100 trials** | | | | | | | | | | | | |
| --- | --- | --- | --- | --- | --- | --- | --- | --- | --- | --- | --- | --- | --- |
| **Participants** | **Congruent cues** | **Two most predictive cues** | **Equal weight** | **Hierarchical** | **Multicue** | **One-cue-1000** | **One-cue-0001** | **Singleton strong** | **Singleton** | **Undetermined** | **One-cue-0100** | **One-cue-0010** | **Number of scores <0.1** |
| **S433** | 0.0618 | 0.0461 | 0.0740 | 0.0636 | 0.0725 | 0.0880 | 0.0488 | 0.0829 | 0.1005 | 0.1311 | 0.2699 | 0.2759 | **8** |
| **S434** | 0.0476 | 0.0259 | 0.0300 | 0.0184 | 0.0214 | 0.0273 | 0.0690 | 0.1246 | 0.1171 | 0.1620 | 0.2806 | 0.2759 | **7** |
| **S435** | 0.0713 | 0.0401 | 0.0681 | 0.0529 | 0.0583 | 0.0428 | 0.0820 | 0.0972 | 0.1100 | 0.1347 | 0.2961 | 0.2568 | **8** |
| **S436** | 0.0809 | 0.1162 | 0.1180 | 0.1136 | 0.1225 | 0.1189 | 0.1581 | 0.1222 | 0.1195 | 0.0847 | 0.1724 | 0.2283 | **2** |
| **S437** | 0.1011 | 0.0235 | 0.0942 | 0.0458 | 0.0690 | 0.0820 | 0.0095 | 0.1638 | 0.1861 | 0.2012 | 0.3163 | 0.3627 | **6** |
| **S438** | 0.0713 | 0.0544 | 0.0835 | 0.0707 | 0.0797 | 0.0856 | 0.0678 | 0.0627 | 0.0791 | 0.1109 | 0.2818 | 0.2878 | **9** |
| **S439** | 0.0761 | 0.1685 | 0.1251 | 0.1611 | 0.1700 | 0.1760 | 0.2057 | 0.0913 | 0.0838 | 0.0645 | 0.0939 | 0.2378 | **5** |
| **S440** | 0.0333 | 0.0425 | 0.0360 | 0.0422 | 0.0416 | 0.0642 | 0.0654 | 0.1032 | 0.1029 | 0.1299 | 0.2247 | 0.1879 | **7** |
| **S441** | 0.0464 | 0.0829 | 0.0657 | 0.0862 | 0.0880 | 0.0987 | 0.1118 | 0.1281 | 0.1314 | 0.1335 | 0.1237 | 0.2438 | **6** |
| **S443** | 0.0547 | 0.0318 | 0.0443 | 0.0196 | 0.0273 | 0.0571 | 0.0511 | 0.1364 | 0.1243 | 0.1846 | 0.2556 | 0.3044 | **7** |
| **S444** | 0.0547 | 0.0627 | 0.0681 | 0.0731 | 0.0820 | 0.0880 | 0.0820 | 0.0829 | 0.0933 | 0.0990 | 0.1819 | 0.2545 | **10** |
| **S445** | 0.0678 | 0.0496 | 0.0181 | 0.0434 | 0.0369 | 0.0595 | 0.0844 | 0.1590 | 0.1528 | 0.1965 | 0.2652 | 0.1998 | **7** |
| **S447** | 0.0535 | 0.0758 | 0.0919 | 0.0791 | 0.0916 | 0.0809 | 0.1153 | 0.0889 | 0.0922 | 0.0835 | 0.2414 | 0.1855 | **9** |
| **S448** | 0.0583 | 0.0544 | 0.0312 | 0.0636 | 0.0690 | 0.0725 | 0.0809 | 0.1186 | 0.1278 | 0.1347 | 0.2164 | 0.1724 | **7** |
| **S449** | 0.0606 | 0.1257 | 0.1204 | 0.1254 | 0.1344 | 0.1498 | 0.1463 | 0.0996 | 0.0993 | 0.0728 | 0.1201 | 0.2426 | **4** |
| **S451** | 0.0357 | 0.0746 | 0.0788 | 0.0803 | 0.0999 | 0.1296 | 0.0642 | 0.1150 | 0.1207 | 0.1418 | 0.2545 | 0.1724 | **6** |
| **S452** | 0.0511 | 0.0330 | 0.0300 | 0.0208 | 0.0202 | 0.0428 | 0.0678 | 0.1329 | 0.1207 | 0.1596 | 0.2818 | 0.1974 | **7** |
| **S453** | 0.0749 | 0.1353 | 0.1061 | 0.1457 | 0.1510 | 0.1641 | 0.1510 | 0.0877 | 0.0981 | 0.0609 | 0.1891 | 0.1332 | **4** |
| **S454** | 0.0392 | 0.0782 | 0.0633 | 0.0707 | 0.0916 | 0.0975 | 0.1034 | 0.1008 | 0.0933 | 0.1168 | 0.1653 | 0.2354 | **7** |
| **S455** | 0.0737 | 0.1257 | 0.1561 | 0.1338 | 0.1463 | 0.1260 | 0.1700 | 0.0734 | 0.0815 | 0.0681 | 0.2913 | 0.1950 | **4** |
|  |  |  |  |  |  |  |  |  |  |  |  |  |  |
| **Moyenne** | 0.0607 | 0.0724 | 0.0751 | 0.0755 | 0.0837 | 0.0926 | 0.0967 | 0.1086 | 0.1117 | 0.1235 | 0.2261 | 0.2325 | **6.50** |
| **1-Moyenne** | 0.9393 | 0.9276 | 0.9249 | 0.9245 | 0.9163 | 0.9074 | 0.9033 | 0.8914 | 0.8883 | 0.8765 | 0.7739 | 0.7675 |  |
| **SD** | 0.0168 | 0.0414 | 0.0375 | 0.0417 | 0.0439 | 0.0408 | 0.0483 | 0.0271 | 0.0258 | 0.0433 | 0.0653 | 0.0539 | **1.96** |
| Green cells represent the strategy with the lowest score for each participant | | | | | | | | | | | | | |
| Orange cells represent the strategies that have a score below the 0.1 criterion for each participant | | | | | | | | | | | | | |

**Table S2.2.** *Scores of each strategy for all the participants that passed the task above chance level across the first 50 training trials. Number of strategies with a score inferior to 0.1, the criterion considered by Gluck et al (2002) to select possible strategies used to solve the task.*

|  | **First 50 trials** | | | | | | | | | | | | |
| --- | --- | --- | --- | --- | --- | --- | --- | --- | --- | --- | --- | --- | --- |
| **Participants** | **Congruent cues** | **Two most predictive cues** | **Equal weight** | **Hierarchical** | **Multicue** | **One-cue-1000** | **One-cue-0001** | **Singleton strong** | **Singleton** | **Undetermined** | **One-cue-0100** | **One-cue-0010** | **Number of scores <0.1** |
| **S433** | 0.0437 | 0.0520 | 0.0465 | 0.0595 | 0.0595 | 0.0743 | 0.0743 | 0.0864 | 0.0939 | 0.1162 | 0.1524 | 0.1710 | **9** |
| **S434** | 0.0550 | 0.0453 | 0.0356 | 0.0399 | 0.0474 | 0.0474 | 0.0991 | 0.0948 | 0.0894 | 0.1466 | 0.3147 | 0.1897 | **9** |
| **S435** | 0.0618 | 0.0869 | 0.0833 | 0.0896 | 0.0860 | 0.0717 | 0.1326 | 0.1030 | 0.1057 | 0.1317 | 0.2975 | 0.1254 | **6** |
| **S436** | 0.0660 | 0.1189 | 0.1233 | 0.1111 | 0.1250 | 0.1250 | 0.1563 | 0.1207 | 0.1128 | 0.1111 | 0.1389 | 0.1910 | **1** |
| **S437** | 0.1689 | 0.0338 | 0.1633 | 0.0867 | 0.1171 | 0.1216 | 0.0045 | 0.2162 | 0.2691 | 0.2410 | 0.3874 | 0.4865 | **3** |
| **S438** | 0.0996 | 0.1228 | 0.1571 | 0.1305 | 0.1327 | 0.1327 | 0.1460 | 0.0454 | 0.0531 | 0.1128 | 0.3274 | 0.4204 | **3** |
| **S439** | 0.1854 | 0.1960 | 0.1801 | 0.1960 | 0.1992 | 0.1992 | 0.2203 | 0.1314 | 0.1314 | 0.0339 | 0.1483 | 0.3686 | **1** |
| **S440** | 0.0243 | 0.0866 | 0.0623 | 0.0827 | 0.0895 | 0.1012 | 0.1051 | 0.0963 | 0.0924 | 0.1138 | 0.1089 | 0.2490 | **7** |
| **S441** | 0.0947 | 0.1178 | 0.1057 | 0.1244 | 0.1366 | 0.1278 | 0.1586 | 0.1861 | 0.1927 | 0.1663 | 0.1189 | 0.2907 | **1** |
| **S443** | 0.0734 | 0.0270 | 0.0344 | 0.0232 | 0.0260 | 0.0260 | 0.0520 | 0.1301 | 0.1264 | 0.1905 | 0.1487 | 0.4535 | **7** |
| **S444** | 0.0587 | 0.0974 | 0.0634 | 0.0915 | 0.0845 | 0.1221 | 0.1268 | 0.1279 | 0.1221 | 0.1092 | 0.1455 | 0.2300 | **5** |
| **S445** | 0.0466 | 0.0233 | 0.0224 | 0.0260 | 0.0215 | 0.0251 | 0.0323 | 0.1452 | 0.1478 | 0.1783 | 0.2222 | 0.2115 | **7** |
| **S447** | 0.0610 | 0.1301 | 0.1636 | 0.1362 | 0.1789 | 0.1545 | 0.1789 | 0.0427 | 0.0488 | 0.0589 | 0.2520 | 0.2154 | **4** |
| **S448** | 0.0716 | 0.1474 | 0.0780 | 0.1635 | 0.1709 | 0.1581 | 0.1667 | 0.1165 | 0.1325 | 0.1132 | 0.2009 | 0.1410 | **2** |
| **S449** | 0.0796 | 0.1318 | 0.1157 | 0.1318 | 0.1542 | 0.1592 | 0.1692 | 0.0609 | 0.0609 | 0.0261 | 0.2438 | 0.2388 | **4** |
| **S451** | 0.0500 | 0.0721 | 0.0651 | 0.0663 | 0.0698 | 0.1535 | 0.0512 | 0.1186 | 0.1128 | 0.1291 | 0.2884 | 0.1442 | **6** |
| **S452** | 0.0843 | 0.0498 | 0.0589 | 0.0447 | 0.0528 | 0.0569 | 0.0691 | 0.1148 | 0.1098 | 0.1159 | 0.3089 | 0.2195 | **7** |
| **S453** | 0.0556 | 0.1473 | 0.0833 | 0.1546 | 0.1643 | 0.1787 | 0.1787 | 0.0688 | 0.0761 | 0.0471 | 0.1836 | 0.1304 | **5** |
| **S454** | 0.0381 | 0.1038 | 0.0657 | 0.0900 | 0.0975 | 0.1102 | 0.1356 | 0.0996 | 0.0858 | 0.0932 | 0.1271 | 0.1568 | **7** |
| **S455** | 0.1196 | 0.1370 | 0.1576 | 0.1565 | 0.1609 | 0.1478 | 0.1739 | 0.0315 | 0.0511 | 0.0283 | 0.2696 | 0.2609 | **3** |
|  |  |  |  |  |  |  |  |  |  |  |  |  |  |
| **Moyenne** | 0.0769 | 0.0964 | 0.0933 | 0.1002 | 0.1087 | 0.1146 | 0.1216 | 0.1068 | 0.1107 | 0.1132 | 0.2193 | 0.2447 | **4.85** |
| **1-Moyenne** | 0.9231 | 0.9036 | 0.9067 | 0.8998 | 0.8913 | 0.8854 | 0.8784 | 0.8932 | 0.8893 | 0.8868 | 0.7807 | 0.7553 |  |
| **SD** | 0.0410 | 0.0473 | 0.0493 | 0.0486 | 0.0530 | 0.0500 | 0.0581 | 0.0457 | 0.0516 | 0.0562 | 0.0830 | 0.1078 | **2.56** |
| Green cells represent the strategy with the lowest score for each participant                    Green cells represent the strategy with the lowest score for each participant                    Green cells represent the strategy with the lowest score for each participant | | | | | | | | | | | | | |
| Orange cells represent the strategies that have a score below the 0.1 criterion for each participant | | | | | | | | | | | | | |

**Table S2.3.** *Scores of each strategy for all the participants that passed the task above chance level across the last 50 training trials. Number of strategies with a score inferior to 0.1, the criterion considered by Gluck et al (2002) to select possible strategies used to solve the task.*

|  | **Last 50 trials** | | | | | | | | | | | | |
| --- | --- | --- | --- | --- | --- | --- | --- | --- | --- | --- | --- | --- | --- |
| **Participants** | **Congruent cues** | **Two most predictive cues** | **Equal weight** | **Hierarchical** | **Multicue** | **One-cue-1000** | **One-cue-0001** | **Singleton strong** | **Singleton** | **Undetermined** | **One-cue-0100** | **One-cue-0010** | **Number of scores <0.1** |
| **S433** | 0.0877 | 0.0517 | 0.1118 | 0.0793 | 0.1010 | 0.1058 | 0.0529 | 0.0793 | 0.1070 | 0.1394 | 0.4135 | 0.3846 | **5** |
| **S434** | 0.0628 | 0.0240 | 0.0502 | 0.0148 | 0.0137 | 0.0274 | 0.0502 | 0.1929 | 0.1838 | 0.2135 | 0.2694 | 0.2694 | **7** |
| **S435** | 0.1155 | 0.0136 | 0.0842 | 0.0353 | 0.0598 | 0.0543 | 0.0435 | 0.1223 | 0.1440 | 0.2011 | 0.2935 | 0.5598 | **6** |
| **S436** | 0.1459 | 0.1541 | 0.1635 | 0.1703 | 0.1892 | 0.1730 | 0.2162 | 0.1514 | 0.1676 | 0.0824 | 0.2757 | 0.3081 | **1** |
| **S437** | 0.0579 | 0.0290 | 0.0601 | 0.0354 | 0.0515 | 0.0773 | 0.0300 | 0.1127 | 0.1191 | 0.1685 | 0.2918 | 0.2618 | **7** |
| **S438** | 0.1018 | 0.0475 | 0.0735 | 0.0679 | 0.0860 | 0.1086 | 0.0452 | 0.1584 | 0.1787 | 0.1867 | 0.3122 | 0.2398 | **5** |
| **S439** | 0.0422 | 0.2294 | 0.1775 | 0.2167 | 0.2353 | 0.2471 | 0.2706 | 0.1343 | 0.1216 | 0.1441 | 0.0784 | 0.2078 | **2** |
| **S440** | 0.1042 | 0.0373 | 0.0537 | 0.0362 | 0.0263 | 0.0965 | 0.0526 | 0.1579 | 0.1568 | 0.1974 | 0.3991 | 0.2456 | **6** |
| **S441** | 0.0413 | 0.0894 | 0.0562 | 0.0929 | 0.0780 | 0.1009 | 0.1193 | 0.1170 | 0.1204 | 0.1491 | 0.1422 | 0.2431 | **5** |
| **S443** | 0.0596 | 0.0631 | 0.0584 | 0.0339 | 0.0374 | 0.1542 | 0.0607 | 0.1600 | 0.1308 | 0.1939 | 0.3879 | 0.1589 | **6** |
| **S444** | 0.0878 | 0.0743 | 0.1047 | 0.0946 | 0.1126 | 0.1081 | 0.0811 | 0.0721 | 0.0923 | 0.1284 | 0.2613 | 0.3108 | **6** |
| **S445** | 0.1148 | 0.1339 | 0.0306 | 0.0931 | 0.0867 | 0.1684 | 0.2143 | 0.2079 | 0.1671 | 0.2449 | 0.2806 | 0.2296 | **3** |
| **S447** | 0.1159 | 0.1212 | 0.1395 | 0.1202 | 0.1159 | 0.1116 | 0.1502 | 0.1964 | 0.1953 | 0.1384 | 0.3133 | 0.2361 | **0** |
| **S448** | 0.0952 | 0.0199 | 0.0397 | 0.0345 | 0.0335 | 0.0418 | 0.0879 | 0.1402 | 0.1548 | 0.1621 | 0.2552 | 0.2343 | **7** |
| **S449** | 0.0554 | 0.1921 | 0.1946 | 0.1913 | 0.1946 | 0.2081 | 0.2047 | 0.1577 | 0.1569 | 0.1493 | 0.0470 | 0.2886 | **2** |
| **S451** | 0.0424 | 0.0902 | 0.1011 | 0.1054 | 0.1348 | 0.1391 | 0.0826 | 0.1359 | 0.1511 | 0.1848 | 0.2261 | 0.2435 | **3** |
| **S452** | 0.0460 | 0.0448 | 0.0261 | 0.0224 | 0.0100 | 0.0597 | 0.0995 | 0.1791 | 0.1567 | 0.2301 | 0.2736 | 0.1990 | **7** |
| **S453** | 0.1281 | 0.1643 | 0.1591 | 0.1756 | 0.1777 | 0.1860 | 0.1736 | 0.1343 | 0.1457 | 0.0888 | 0.2273 | 0.1612 | **1** |
| **S454** | 0.0584 | 0.0530 | 0.0660 | 0.0530 | 0.0866 | 0.0866 | 0.0649 | 0.1245 | 0.1245 | 0.1634 | 0.2121 | 0.3333 | **7** |
| **S455** | 0.0708 | 0.1491 | 0.1996 | 0.1481 | 0.1803 | 0.1373 | 0.2103 | 0.1373 | 0.1363 | 0.1341 | 0.3391 | 0.1803 | **1** |
|  |  |  |  |  |  |  |  |  |  |  |  |  |  |
| **Moyenne** | 0.0817 | 0.0891 | 0.0975 | 0.0910 | 0.1005 | 0.1196 | 0.1155 | 0.1436 | 0.1455 | 0.1650 | 0.2650 | 0.2648 | **4.35** |
| **1-Moyenne** | 0.9183 | 0.9109 | 0.9025 | 0.9090 | 0.8995 | 0.8804 | 0.8845 | 0.8564 | 0.8545 | 0.8350 | 0.7350 | 0.7352 |  |
| **SD** | 0.0321 | 0.0629 | 0.0561 | 0.0615 | 0.0664 | 0.0572 | 0.0739 | 0.0352 | 0.0266 | 0.0425 | 0.0947 | 0.0895 | **2.46** |
| Green cells represent the strategy with the lowest score for each participant | | | | | | | | | | | | | |
| Orange cells represent the strategies that have a score below the 0.1 criterion for each participant | | | | | | | | | | | | | |

| **Table S3.** *Correspondence between the performance of the group of participants for each individual pattern and the performance predicted by each strategy. A green check mark indicates that participants performed as predicted by the strategy, whereas a red cross indicates that participants did not perform as predicted by the strategy. Only the performance predicted by the hierarchical strategy was entirely consistent with the performance of the group of young adult participants for all patterns.* |
| --- |

| **Strategies** | **Patterns** | | | | | | | | | | | | | | |
| --- | --- | --- | --- | --- | --- | --- | --- | --- | --- | --- | --- | --- | --- | --- | --- |
|  | | **0001** | **0010** | **0011** | **0100** | **0101** | **0110** | **0111** | **1000** | **1001** | **1010** | **1011** | **1100** | **1101** | **1110** |
| **Multicue** | | **✓** | **✓** | **✓** | **✓** | **✓** | **-** | **✓** | **✓** | **-** | **✓** | **🗶** | **✓** | **🗶** | **✓** |
| **Hierarchical** | | **✓** | **✓** | **✓** | **✓** | **✓** | **-** | **✓** | **✓** | **-** | **✓** | **✓** | **✓** | **✓** | **✓** |
| **Equal weight** | | **✓** | **✓** | **✓** | **✓** | **🗶** | **-** | **✓** | **✓** | **-** | **🗶** | **🗶** | **✓** | **🗶** | **✓** |
| **One-cue-1000** | | **✓** | **✓** | **✓** | **🗶** | **✓** | **-** | **✓** | **✓** | **-** | **✓** | **🗶** | **✓** | **🗶** | **✓** |
| **Two most predictive cues** | | **✓** | **🗶** | **✓** | **🗶** | **✓** | **-** | **✓** | **✓** | **-** | **✓** | **✓** | **✓** | **✓** | **✓** |
| **One-cue-0001** | | **✓** | **🗶** | **✓** | **✓** | **✓** | **-** | **✓** | **✓** | **-** | **✓** | **🗶** | **✓** | **🗶** | **✓** |
| **Congruent cues** | | **✓** | **✓** | **✓** | **✓** | **🗶** | **-** | **🗶** | **✓** | **-** | **🗶** | **✓** | **✓** | **✓** | **🗶** |
| **One-cue-0010** | | **🗶** | **✓** | **✓** | **✓** | **🗶** | **-** | **✓** | **✓** | **-** | **🗶** | **🗶** | **✓** | **🗶** | **🗶** |
| **One-cue-0100** | | **✓** | **✓** | **✓** | **✓** | **🗶** | **-** | **🗶** | **🗶** | **-** | **🗶** | **🗶** | **✓** | **🗶** | **✓** |
| **Singleton** | | **✓** | **✓** | **🗶** | **✓** | **🗶** | **-** | **🗶** | **✓** | **-** | **🗶** | **✓** | **🗶** | **✓** | **🗶** |
| **Singleton strong** | | **✓** | **🗶** | **🗶** | **🗶** | **🗶** | **-** | **🗶** | **✓** | **-** | **🗶** | **✓** | **🗶** | **✓** | **🗶** |
| **Undetermined** | | **🗶** | **🗶** | **🗶** | **🗶** | **🗶** | **-** | **🗶** | **🗶** | **-** | **🗶** | **✓** | **🗶** | **✓** | **🗶** |
| The patterns including either the two highly predictive cues (1001) or the two less predictive cues (0110) are not associated with a predictable answer but are nonetheless listed for the sake of completeness. The overall group performance was above chance for all other patterns, except for the two patterns that included the two highly predictive cues and one of the two less predictive cues (patterns 1011 and 1101). | | | | | | | | | | | | | | | |
