# Supplementary material for: What Is the Weather Prediction Task Good for? A New Analysis of Learning Strategies Reveals How Young Adults Solve the Task
Source: Front Psychol. 2022 Jun 13;13:886339. doi: 10.3389/fpsyg.2022.886339 (PMC9234396; doi:10.3389/fpsyg.2022.886339)

**FIGURE S3.** Individual performance of 15 young healthy adults in the 200-trial-WPT (closed circles: women; open circles: men). (A) Number of correct choices during training trials 1-100 with various combinations of cues and feedback. The black line represents the number of correct choices (56/93) defined as statistically different from chance at the individual level (χ^2^_(1)_ = 3.882, p = .049). (B) Number of correct choices during training trials 101-200 with various combinations of cues and feedback. The black line represents the number of correct choices (56/93) defined as statistically different from chance at the individual level (χ^2^_(1)_ = 3.882, p = .049). (C) Number of correct choices during training trials 1-200 with various combinations of cues and feedback. The black line represents the number of correct choices (107/186) defined as statistically different from chance at the individual level (χ^2^_(1)_ = 4.215, p = .040). (D) Number of correct choices during the four test trials with individual cues and no feedback. Although we did not impose a response time inferior to 5 seconds as was originally done, our participants' average response time (M = 2.27; SD = 0.75 was similar to that observed in previous studies).


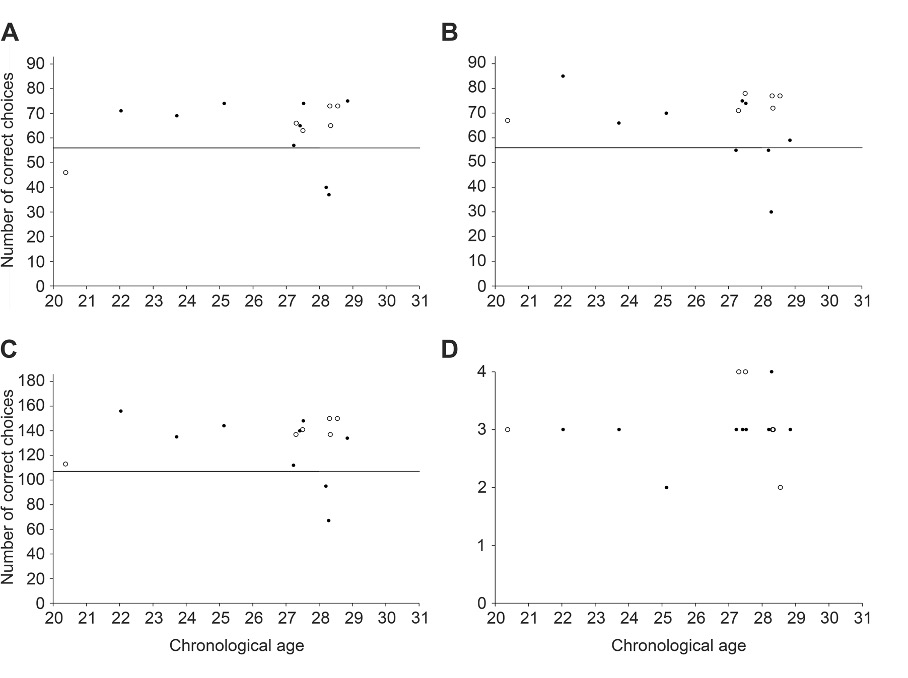


**FIGURE S4.** Means of fitted scores (± SE) (A) across the 200 trials; (B) across the two blocks of 100 trials for the group of 11 young adults who performed the 200-trial-WPT above chance level. Note that in order to make the graphical representation more intuitive, the y axis represents 1-score generated by the model. Therefore, the higher the value, the more likely was a given strategy to be used by the group of participants. To simplify comparisons, strategies are listed in ranking order from the most likely used (congruent cues) to the least likely used (one less predictive cue) across the 100-trial-WPT.


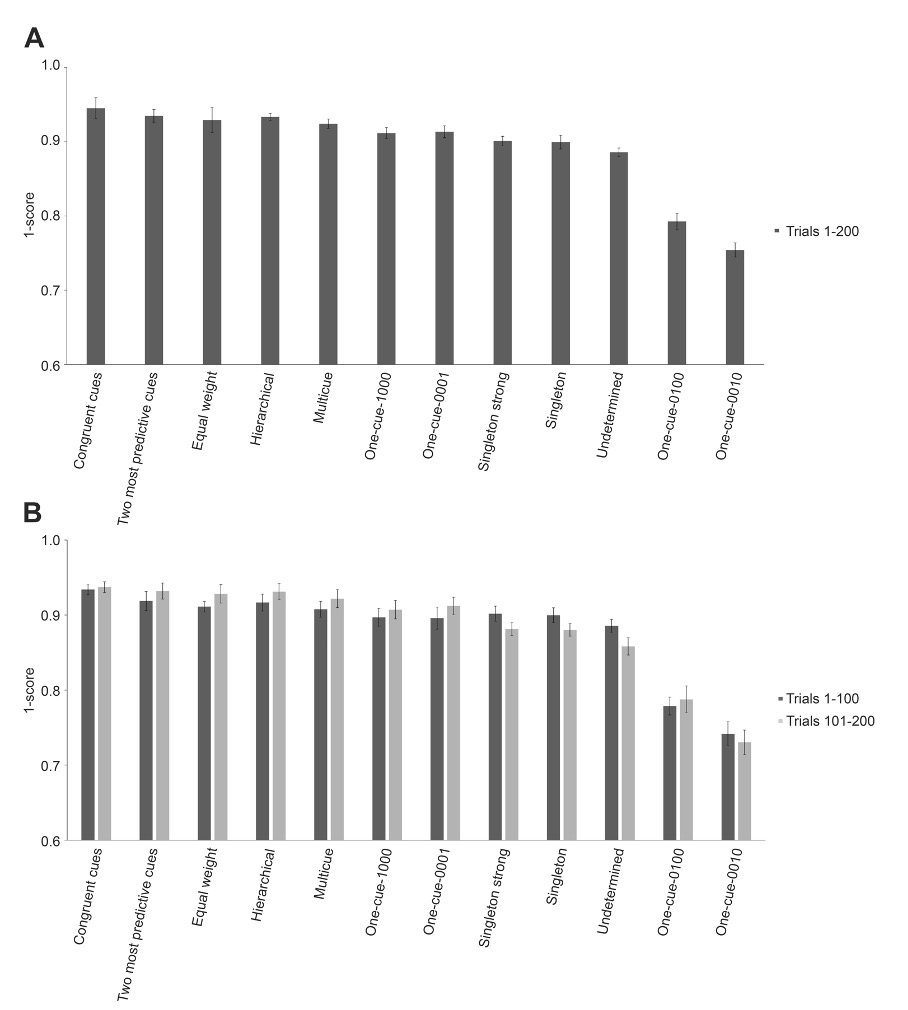


**FIGURE S5. (**A) Normalized number of correct choices (NCC) mean (± SE) for each category of patterns for the participants who performed the task above chance level during the training phase of the 200-trial-WPT; (B) Number of correct choices (NCC) mean (± SE) for each category of patterns for the participants who performed the task above chance level during the testing phase.


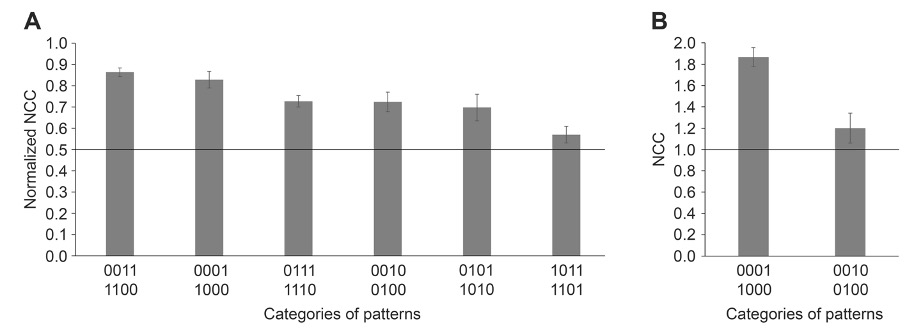


**FIGURE S6**. Normalized number of correct choices (NCC) mean (± SE) for each category of patterns for the participants who performed the task above chance level during the first 100 trials of the training phase.


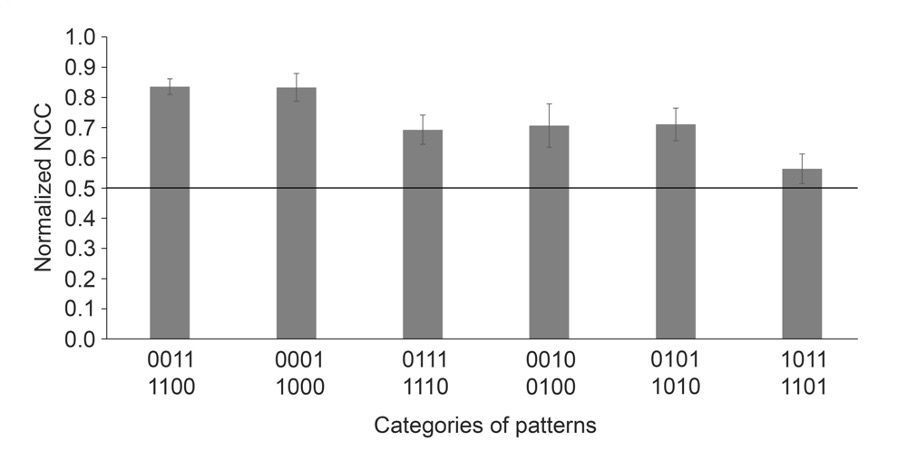


**FIGURE S7.** Normalized number of correct choices (NCC) mean (± SE) for each category of patterns for the participants who performed the task above chance level during the last 100 trials of the training phase


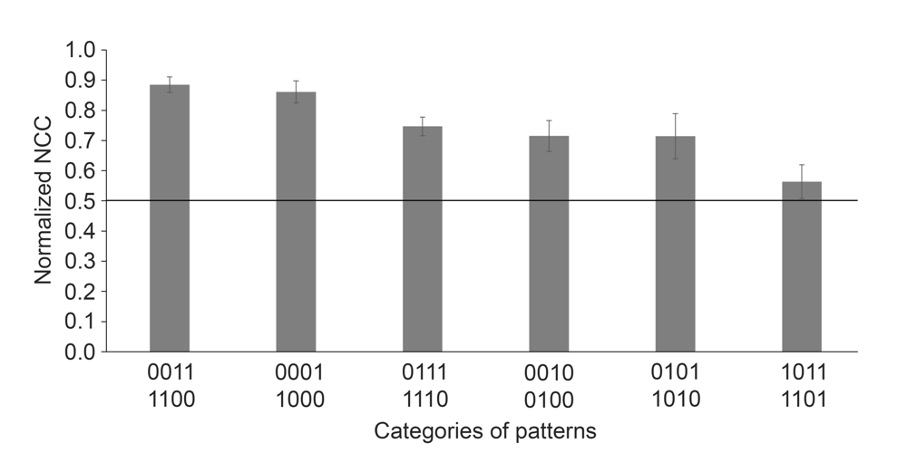

Supplement: Supplementary file 3 [file Data_Sheet_3.docx]
