# Supplementary material for: What Is the Weather Prediction Task Good for? A New Analysis of Learning Strategies Reveals How Young Adults Solve the Task
Source: Front Psychol. 2022 Jun 13;13:886339. doi: 10.3389/fpsyg.2022.886339 (PMC9234396; doi:10.3389/fpsyg.2022.886339)
Supplement: Supplementary file 4 [file Data_Sheet_4.docx]

**Table S4.1.** *Scores (actual response - theoretical response; Gluck et al., 2002) for all the strategies for each participant who performed the task above chance level across the 200 training trials. Number of strategies with a score inferior to 0.1, the criterion considered by Gluck et al (2002) to select possible strategies used to solve the task.*

|  | **200 trials** | | | | | | | | | | | | |
| --- | --- | --- | --- | --- | --- | --- | --- | --- | --- | --- | --- | --- | --- |
| **Participants** | **Congruent cues** | **Two most predictive cues** | **Equal weight** | **Hierarchical** | **Multicue** | **One-cue-1000** | **One-cue-0001** | **Singleton strong** | **Singleton** | **Undetermined** | **One-cue-0100** | **One-cue-0010** | **Number of scores <0.1** |
| **S518** | 0.0648 | 0.0936 | 0.0705 | 0.0927 | 0.0993 | 0.1207 | 0.1112 | 0.0889 | 0.0880 | 0.0782 | 0.1908 | 0.1778 | **8** |
| **S520** | 0.0806 | 0.0517 | 0.0737 | 0.0669 | 0.0728 | 0.0693 | 0.0788 | 0.0809 | 0.0960 | 0.0862 | 0.2048 | 0.2857 | **10** |
| **S521** | 0.0244 | 0.0443 | 0.0532 | 0.0452 | 0.0434 | 0.0482 | 0.0850 | 0.1162 | 0.1171 | 0.1590 | 0.2348 | 0.2206 | **7** |
| **S523** | 0.0743 | 0.0300 | 0.0794 | 0.0398 | 0.0583 | 0.0702 | 0.0345 | 0.0877 | 0.0975 | 0.1359 | 0.3068 | 0.3222 | **9** |
| **S524** | 0.0595 | 0.0271 | 0.0586 | 0.0345 | 0.0529 | 0.0696 | 0.0291 | 0.1293 | 0.1367 | 0.1507 | 0.2289 | 0.2860 | **7** |
| **S525** | 0.0568 | 0.0927 | 0.0642 | 0.0853 | 0.0883 | 0.0942 | 0.1359 | 0.1183 | 0.1109 | 0.1023 | 0.1787 | 0.1656 | **6** |
| **S526** | 0.0523 | 0.0383 | 0.0728 | 0.0446 | 0.0559 | 0.0595 | 0.0618 | 0.1079 | 0.1141 | 0.1079 | 0.2188 | 0.2473 | **7** |
| **S527** | 0.0178 | 0.1127 | 0.0633 | 0.1058 | 0.1076 | 0.1326 | 0.1373 | 0.0865 | 0.0797 | 0.1347 | 0.1540 | 0.1980 | **4** |
| **S529** | 0.0339 | 0.0467 | 0.0514 | 0.0398 | 0.0488 | 0.0595 | 0.0785 | 0.0716 | 0.0648 | 0.1091 | 0.2485 | 0.2224 | **9** |
| **S532** | 0.0746 | 0.0815 | 0.1058 | 0.0865 | 0.1067 | 0.1246 | 0.0829 | 0.1159 | 0.1210 | 0.1159 | 0.1555 | 0.3374 | **4** |
| **S533** | 0.0642 | 0.0984 | 0.0859 | 0.0916 | 0.0993 | 0.1243 | 0.1171 | 0.0865 | 0.0797 | 0.0758 | 0.1599 | 0.2408 | **8** |
| **Moyenne** | 0.0548 | 0.0652 | 0.0708 | 0.0666 | 0.0757 | 0.0884 | 0.0866 | 0.0991 | 0.1005 | 0.1141 | 0.2074 | 0.2458 | **7.18** |
| **1-Moyenne** | 0.9452 | 0.9348 | 0.9292 | 0.9334 | 0.9243 | 0.9116 | 0.9134 | 0.9009 | 0.8995 | 0.8859 | 0.7926 | 0.7542 |  |
| **SD** | 0.0467 | 0.0283 | 0.0566 | 0.0157 | 0.0209 | 0.0250 | 0.0265 | 0.0215 | 0.0309 | 0.0189 | 0.0365 | 0.0316 | **1.94** |
| Green cells represent the strategy with the lowest score for each participant | | | | | | | | | | | | | |
| Orange cells represent the strategies that have a score below the 0.1 criterion for each participant | | | | | | | | | | | | | |

**Table S4.2.** *Scores of each strategy for all the participants that passed the task above chance level across the first 100 training trials. Number of strategies with a score inferior to 0.1, the criterion considered by Gluck et al (2002) to select possible strategies used to solve the task.*

|  | **Trials 1-100** | | | | | | | | | | | | |
| --- | --- | --- | --- | --- | --- | --- | --- | --- | --- | --- | --- | --- | --- |
| **Participants** | **Congruent cues** | **Two most predictive cues** | **Equal weight** | **Hierarchical** | **Multicue** | **One-cue-1000** | **One-cue-0001** | **Singleton strong** | **Singleton** | **Undetermined** | **One-cue-0100** | **One-cue-0010** | **Number of scores <0.1** |
| **S518** | 0.0785 | 0.1174 | 0.1121 | 0.1278 | 0.1332 | 0.1463 | 0.1332 | 0.0294 | 0.0398 | 0.0669 | 0.1926 | 0.3127 | **4** |
| **S520** | 0.0892 | 0.0699 | 0.1026 | 0.0838 | 0.0927 | 0.0797 | 0.1046 | 0.0829 | 0.0969 | 0.0669 | 0.1974 | 0.2913 | **8** |
| **S521** | 0.0392 | 0.0913 | 0.1109 | 0.0993 | 0.1023 | 0.0892 | 0.1379 | 0.1162 | 0.1243 | 0.1537 | 0.2687 | 0.2128 | **4** |
| **S523** | 0.0642 | 0.0473 | 0.0859 | 0.0517 | 0.0690 | 0.0820 | 0.0571 | 0.0675 | 0.0719 | 0.1156 | 0.2735 | 0.3246 | **9** |
| **S524** | 0.0785 | 0.0235 | 0.0645 | 0.0446 | 0.0618 | 0.0654 | 0.0262 | 0.1269 | 0.1480 | 0.1537 | 0.2663 | 0.2889 | **7** |
| **S525** | 0.0963 | 0.1341 | 0.1061 | 0.1219 | 0.1272 | 0.1498 | 0.1629 | 0.1317 | 0.1195 | 0.0942 | 0.2342 | 0.1570 | **2** |
| **S526** | 0.0571 | 0.0330 | 0.0657 | 0.0434 | 0.0488 | 0.0618 | 0.0488 | 0.1162 | 0.1266 | 0.1216 | 0.2342 | 0.2402 | **7** |
| **S527** | 0.0297 | 0.1567 | 0.1204 | 0.1492 | 0.1570 | 0.1772 | 0.1807 | 0.0758 | 0.0684 | 0.1240 | 0.1831 | 0.2200 | **3** |
| **S529** | 0.0321 | 0.0532 | 0.0479 | 0.0410 | 0.0535 | 0.0618 | 0.0892 | 0.0829 | 0.0707 | 0.1204 | 0.2366 | 0.2212 | **9** |
| **S532** | 0.0713 | 0.0794 | 0.0847 | 0.0779 | 0.0975 | 0.1177 | 0.0856 | 0.1115 | 0.1100 | 0.1168 | 0.1570 | 0.3151 | **6** |
| **S533** | 0.0880 | 0.0877 | 0.0752 | 0.0755 | 0.0713 | 0.1011 | 0.1189 | 0.1388 | 0.1266 | 0.1228 | 0.1902 | 0.2580 | **5** |
| **Moyenne** | 0.0658 | 0.0812 | 0.0887 | 0.0833 | 0.0922 | 0.1029 | 0.1041 | 0.0982 | 0.1003 | 0.1142 | 0.2213 | 0.2584 | **5.82** |
| **1-Moyenne** | 0.9342 | 0.9188 | 0.9113 | 0.9167 | 0.9078 | 0.8971 | 0.8959 | 0.9018 | 0.8997 | 0.8858 | 0.7787 | 0.7416 |  |
| **SD** | 0.0235 | 0.0420 | 0.0235 | 0.0375 | 0.0354 | 0.0397 | 0.0484 | 0.0333 | 0.0333 | 0.0287 | 0.0394 | 0.0530 | **2.40** |
| Green cells represent the strategy with the lowest score for each participant | | | | | | | | | | | | | |
| Orange cells represent the strategies that have a score below the 0.1 criterion for each participant | | | | | | | | | | | | | |

**Table S4.3.** *Scores of each strategy for all the participants that passed the task above chance level across the last 100 training trials. Number of strategies with a score inferior to 0.1, the criterion considered by Gluck et al (2002) to select possible strategies used to solve the task.*

|  | **Trials 101-200** | | | | | | | | | | | | |
| --- | --- | --- | --- | --- | --- | --- | --- | --- | --- | --- | --- | --- | --- |
| **Participants** | **Congruent cues** | **Two most predictive cues** | **Equal weight** | **Hierarchical** | **Multicue** | **One-cue-1000** | **One-cue-0001** | **Singleton strong** | **Singleton** | **Undetermined** | **One-cue-0100** | **One-cue-0010** | **Number of scores <0.1** |
| **S518** | 0.0416 | 0.0603 | 0.0193 | 0.0482 | 0.0559 | 0.0856 | 0.0797 | 0.1388 | 0.1266 | 0.1763 | 0.1795 | 0.2259 | **7** |
| **S520** | 0.0939 | 0.0556 | 0.0669 | 0.0719 | 0.0749 | 0.0809 | 0.0749 | 0.1008 | 0.1171 | 0.1275 | 0.2342 | 0.3020 | **7** |
| **S521** | 0.0440 | 0.0318 | 0.0300 | 0.0256 | 0.0190 | 0.0416 | 0.0666 | 0.1507 | 0.1445 | 0.1989 | 0.2354 | 0.2628 | **7** |
| **S523** | 0.0939 | 0.0223 | 0.0823 | 0.0375 | 0.0571 | 0.0678 | 0.0214 | 0.1174 | 0.1326 | 0.1656 | 0.3496 | 0.3294 | **7** |
| **S524** | 0.0559 | 0.0461 | 0.0681 | 0.0398 | 0.0595 | 0.0892 | 0.0476 | 0.1471 | 0.1409 | 0.1632 | 0.2069 | 0.2985 | **7** |
| **S525** | 0.0488 | 0.0829 | 0.0538 | 0.0803 | 0.0809 | 0.0702 | 0.1403 | 0.1364 | 0.1338 | 0.1418 | 0.1546 | 0.2057 | **6** |
| **S526** | 0.0666 | 0.0627 | 0.0990 | 0.0648 | 0.0820 | 0.0761 | 0.0939 | 0.1186 | 0.1207 | 0.1133 | 0.2224 | 0.2735 | **7** |
| **S527** | 0.0309 | 0.0936 | 0.0312 | 0.0874 | 0.0832 | 0.1130 | 0.1189 | 0.1222 | 0.1159 | 0.1703 | 0.1498 | 0.2010 | **5** |
| **S529** | 0.0428 | 0.0473 | 0.0621 | 0.0458 | 0.0511 | 0.0642 | 0.0749 | 0.0675 | 0.0660 | 0.1049 | 0.2675 | 0.2307 | **9** |
| **S532** | 0.0939 | 0.0996 | 0.1430 | 0.1112 | 0.1320 | 0.1474 | 0.0963 | 0.1364 | 0.1480 | 0.1311 | 0.1700 | 0.3757 | **3** |
| **S533** | 0.0773 | 0.1460 | 0.1335 | 0.1445 | 0.1641 | 0.1843 | 0.1522 | 0.0710 | 0.0696 | 0.0657 | 0.1665 | 0.2604 | **4** |
| **Moyenne** | 0.0627 | 0.0680 | 0.0717 | 0.0688 | 0.0782 | 0.0927 | 0.0879 | 0.1188 | 0.1196 | 0.1417 | 0.2124 | 0.2696 | **6.27** |
| **1-Moyenne** | 0.9373 | 0.9320 | 0.9283 | 0.9312 | 0.9218 | 0.9073 | 0.9121 | 0.8812 | 0.8804 | 0.8583 | 0.7876 | 0.7304 |  |
| **SD** | 0.0237 | 0.0354 | 0.0404 | 0.0356 | 0.0398 | 0.0410 | 0.0385 | 0.0285 | 0.0277 | 0.0382 | 0.0594 | 0.0539 | **1.68** |
| Green cells represent the strategy with the lowest score for each participant | | | | | | | | | | | | | |
| Orange cells represent the strategies that have a score below the 0.1 criterion for each participant | | | | | | | | | | | | | |

**TABLE S5.** Two-tailed t-tests comparing the performance of the group of participants with chance level for each individual pattern.

| Patterns | First 100 trials | | | | | |
| --- | --- | --- | --- | --- | --- | --- |
|  | Mean | SD | *n* | *t(10)* | *p* | *Cohen's d_z_* |
| 0001 | 7.82 | 2.09 | 9 | 5.268 | <.001** | 1.588 |
| 0010 | 3.55 | 1.44 | 5 | 2.408 | .037** | 0.726 |
| 0011 | 10.45 | 1.63 | 13 | 8.023 | <.001** | 2.419 |
| 0100 | 2.82 | 1.08 | 4 | 2.516 | .031** | 0.758 |
| 0101 | 4.09 | 1.45 | 6 | 2.502 | .031** | 0.754 |
| 0110^a^ | - | - | 3 | - | - | - |
| 0111 | 6.18 | 1.66 | 10 | 2.358 | .040** | 0.711 |
| 1000 | 7.18 | 1.78 | 9 | 5.001 | <.001** | 1.508 |
| 1001^a^ | - | - | 4 | - | - | - |
| 1010 | 5.18 | 1.17 | 7 | 4.777 | <.001** | 1.440 |
| 1011 | 1.91 | 0.94 | 3 | 1.437 | .181 | 0.433 |
| 1100 | 11.27 | 1.56 | 13 | 10.179 | <.001** | 3.069 |
| 1101 | 2.45 | 1.29 | 5 | 0.117 | .910 | 0.035 |
| 1110 | 6.91 | 2.07 | 9 | 3.857 | .003** | 1.163 |
| ^a^ Those patterns do not have any incorrect answer. Two asterisks (**) represent *P* values <.05 for two-tailed, one-sample Student’s *t*-test comparisons. *n* represents the number of presentations of each pattern. | | | | | | |

**TABLE S6.** Two-tailed t-tests comparing the performance of the group of participants with chance level for each individual pattern.

| Patterns | Last 100 trials | | | | | |
| --- | --- | --- | --- | --- | --- | --- |
|  | Mean | SD | *n* | *t(10)* | *p* | *Cohen's d_z_* |
| 0001 | 8.55 | 0.82 | 9 | 16.359 | <.001** | 4.932 |
| 0010 | 3.55 | 1.51 | 5 | 2.300 | .044** | 0.693 |
| 0011 | 11.45 | 1.51 | 13 | 10.900 | <.001** | 3.286 |
| 0100 | 3.09 | 1.04 | 4 | 3.464 | .006** | 1.044 |
| 0101 | 4.00 | 1.95 | 6 | 1.701 | .120 | 0.513 |
| 0110^a^ | - | - | 3 | - | - | - |
| 0111 | 6.73 | 1.35 | 10 | 4.249 | .002** | 1.281 |
| 1000 | 7.09 | 1.92 | 9 | 4.473 | .001** | 1.349 |
| 1001^a^ | - | - | 4 | - | - | - |
| 1010 | 4.91 | 2.07 | 7 | 2.256 | .048** | 0.680 |
| 1011 | 2.09 | 1.04 | 3 | 1.876 | .090* | 0.566 |
| 1100 | 11.73 | 1.35 | 13 | 12.857 | <.001** | 3.877 |
| 1101 | 2.27 | 2.01 | 5 | 0.376 | .715 | 0.113 |
| 1110 | 7.64 | 1.21 | 9 | 8.625 | <.001** | 2.601 |
| ^a^ Those patterns do not have a correct answer. Two asterisks (**) represent *P* values <.05 for two-tailed, one-sample Student’s *t*-test comparisons. One asterisk (*) represents *P* values <.10 for two-tailed, one-sample Student’s *t*-test comparisons. *n* represents the number of presentations of each pattern. | | | | | | |

**TABLE S7.** Two-tailed t-tests comparing the performance of the group of participants with chance level for each individual pattern.

| Patterns | 200 trials | | | | | |
| --- | --- | --- | --- | --- | --- | --- |
|  | Mean | SD | *n* | *t_(10)_* | *p* | *Cohen's d_z_* |
| 0001 | 15.55 | 3.47 | 18 | 6.248 | <.001** | 1.884 |
| 0010 | 7.09 | 2.07 | 10 | 3.348 | .007** | 1.009 |
| 0011 | 21.91 | 2.26 | 26 | 13.096 | <.001** | 3.949 |
| 0100 | 5.91 | 1.22 | 8 | 5.186 | <.001** | 1.564 |
| 0101 | 8.09 | 3.18 | 12 | 2.183 | .054* | 0.658 |
| 0110^a^ | - | - | 6 | - | - | - |
| 0111 | 12.91 | 2.07 | 20 | 4.658 | <.001** | 1.404 |
| 1000 | 14.27 | 3.58 | 18 | 4.884 | <.001** | 1.473 |
| 1001^a^ | - | - | 8 | - | - | - |
| 1010 | 10.09 | 2.51 | 14 | 4.087 | .002** | 1.232 |
| 1011 | 4.00 | 1.55 | 6 | 2.141 | .058* | 0.645 |
| 1100 | 23.00 | 2.45 | 26 | 13.540 | <.001** | 4.082 |
| 1101 | 4.73 | 2.83 | 10 | 0.319 | .756 | 0.096 |
| 1110 | 14.55 | 2.46 | 18 | 7.463 | <.001** | 2.250 |
| ^a^ Those patterns do not have a correct answer. Two asterisks (**) represent *P* values <.05 for two-tailed, one-sample Student’s *t*-test comparisons. One asterisk (*) represents *P* values <.10 for two-tailed, one-sample Student’s *t*-test comparisons. *n* represents the number of presentations of each pattern. | | | | | | |

**Table S8.** P values and Cohen’s dz for the post-hoc comparisons between the different categories of patterns across the first 100 training trials.

|  | Values | 0011  &  1100 | 0001  &  1000 | 0111  &  1110 | 0010  &  0100 | 0101  &  1010 | 1011  &  1101 |
| --- | --- | --- | --- | --- | --- | --- | --- |
| 0011 & 1100 | *p* | - |  |  |  |  |  |
|  | *d_z_* | - |  |  |  |  |  |
| 0001 & 1000 | *p* | .966 | - |  |  |  |  |
|  | *d_z_* | 0.013 | - |  |  |  |  |
| 0111 & 1110 | *p* | .032** | .113 | - |  |  |  |
|  | *d_z_* | 0.752 | 0.524 | - |  |  |  |
| 0010 & 0100 | *p* | .133 | .205 | .880 | - |  |  |
|  | *d_z_* | 0.494 | 0.408 | 0.047 | - |  |  |
| 0101 & 1010 | *p* | .036** | .120 | .767 | .970 | - |  |
|  | *d_z_* | 0.729 | 0.512 | 0.092 | 0.012 | - |  |
| 1011 & 1101 | *p* | <.001** | .006** | .124 | .079* | .102 | - |
|  | *d_z_* | 1.468 | 1.040 | 0.506 | 0.589 | 0.543 | - |
| Two asterisks (**) represent *P* values <.05 for two-tailed, paired *t*-test comparisons. One asterisk (*) represents *P* values <.10 for two-tailed, paired *t*-test comparisons, which would be equivalent to *P* values <.05 for one-tailed comparisons. | | | | | | | |

**Table S9.** P values and Cohen’s dz for the post-hoc comparisons between the different categories of patterns across the last 100 training trials.

|  | Values | 0011  &  1100 | 0001  &  1000 | 0111  &  1110 | 0010  &  0100 | 0101  &  1010 | 1011  &  1101 |
| --- | --- | --- | --- | --- | --- | --- | --- |
| 0011 & 1100 | *p* | - |  |  |  |  |  |
|  | *d_z_* | - |  |  |  |  |  |
| 0001 & 1000 | *p* | .546 | - |  |  |  |  |
|  | *d_z_* | 0.189 | - |  |  |  |  |
| 0111 & 1110 | *p* | .003** | .004** | - |  |  |  |
|  | *d_z_* | 1.171 | 1.132 | - |  |  |  |
| 0010 & 0100 | *p* | .008** | .054* | .773 | - |  |  |
|  | *d_z_* | 0.987 | 0.657 | 0.089 | - |  |  |
| 0101 & 1010 | *p* | .037** | .061* | .373 | .637 | - |  |
|  | *d_z_* | 0.727 | 0.637 | 0.281 | 0.147 | - |  |
| 1011 & 1101 | *p* | <.001** | <.001** | .006** | .008** | .366 | - |
|  | *d_z_* | 1.902 | 2.541 | 1.034 | 1.003 | 0.286 | - |
| Two asterisks (**) represent *P* values <.05 for two-tailed, paired *t*-test comparisons. One asterisk (*) represents *P* values <.10 for two-tailed, paired *t*-test comparisons, which would be equivalent to *P* values <.05 for one-tailed comparisons. | | | | | | | |

**Table S10.** P values and Cohen’s dz for the post-hoc comparisons between the different categories of patterns across the 200 training trials.

|  | Values | 0011  &  1100 | 0001  &  1000 | 0111  &  1110 | 0010  &  0100 | 0101  &  1010 | 1011  &  1101 |
| --- | --- | --- | --- | --- | --- | --- | --- |
| 0011 & 1100 | *p* | - |  |  |  |  |  |
|  | *d_z_* | - |  |  |  |  |  |
| 0001 & 1000 | *p* | .375 | - |  |  |  |  |
|  | *d_z_* | 0.280 | - |  |  |  |  |
| 0111 & 1110 | *p* | .006** | .090* | - |  |  |  |
|  | *d_z_* | 1.053 | 0.566 | - |  |  |  |
| 0010 & 0100 | *p* | .018** | .127 | .966 | - |  |  |
|  | *d_z_* | 0.850 | 0.501 | 0.013 | - |  |  |
| 0101 & 1010 | *p* | .027** | .075* | .618 | .799 | - |  |
|  | *d_z_* | 0.780 | 0.599 | 0.155 | 0.079 | - |  |
| 1011 & 1101 | *p* | <.001** | <.001** | .027** | .005** | .196 | - |
|  | *d_z_* | 1.047 | 1.475 | 0.778 | 1.079 | 0.418 | - |
| Two asterisks (**) represent *P* values <.05 for two-tailed, paired *t*-test comparisons. One asterisk (*) represents *P* values <.10 for two-tailed, paired *t*-test comparisons, which would be equivalent to *P* values <.05 for one-tailed comparisons. | | | | | | | |
